# Combination of Molecular Dynamics Simulations and Machine Learning Reveals Structural Characteristics of Stereochemistry-Specific Interdigitation of Synthetic Monomycoloyl Glycerol Analogs

**DOI:** 10.1021/acs.jcim.5c00615

**Published:** 2025-07-03

**Authors:** Suvi Heinonen, Artturi Koivuniemi, Matthew Davies, Mikko Karttunen, Camilla Foged, Alex Bunker

**Affiliations:** ‡ Drug Research Program, Division of Pharmaceutical Biosciences, Faculty of Pharmacy, 3835 University of Helsinki, Viikinkaari 5 E, P.O. Box 56, 00014 Helsinki, Finland; § Department of Physics and Astronomy, 6221 The University of Western Ontario, 1151 Richmond Street, London, Ontario N6A 3K7, Canada; ∥ Department of Chemistry, 6221 The University of Western Ontario, 1151 Richmond Street, London, Ontario N6A 5B7, Canada; ⊥ Department of Pharmacy, Faculty of Health and Medical Sciences, University of Copenhagen, Universitetsparken 2, 2100 København Ø, Denmark

## Abstract

Synthetic monomycoloyl glycerol (MMG) analogs possess robust immunostimulatory activity and are investigated as adjuvants for subunit vaccines in preclinical and clinical studies. These synthetic lipids consist of a glycerol moiety attached to a corynomycolic acid. Previous experimental studies have shown that the stereochemistry of the lipid acid moiety affects whether the MMG analogs self-assemble into interdigitated or noninterdigitated structures below the main phase transition temperature (*T*_m_). In this study, we elucidated possible thermodynamic mechanisms governing the phase behavior of MMG analogs by exploring their conformations, interactions, and dynamics using a combination of machine learning (ML) and molecular dynamics (MD) simulations. We compared two analogs, MMG-1 and MMG-6, which differ only by the stereochemistry of the lipid acid moiety; the former has a configuration different from the natural MMG, and the latter displays a native-like stereochemistry. Three different membrane states were simulated: (1) a noninterdigitated single bilayer, (2) a noninterdigitated double bilayer, and (3) a fully interdigitated double bilayer. Our results indicate that the propensity for interdigitation of the MMG analogs in a bilayer is linked to the degree to which their hydrocarbon chains are ordered and oriented. This study demonstrates how combining MD simulations with ML can enhance the molecular understanding of lipid-based pharmaceutical formulations.

## Graphical abstract

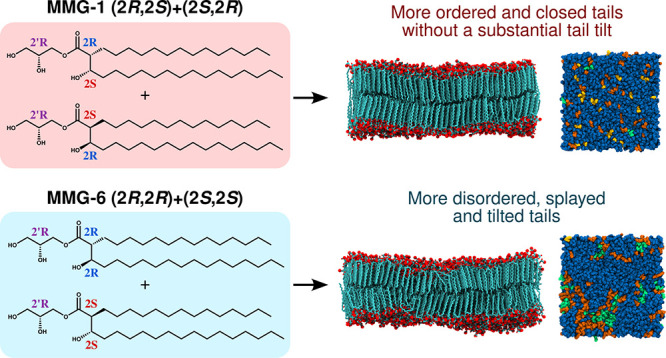

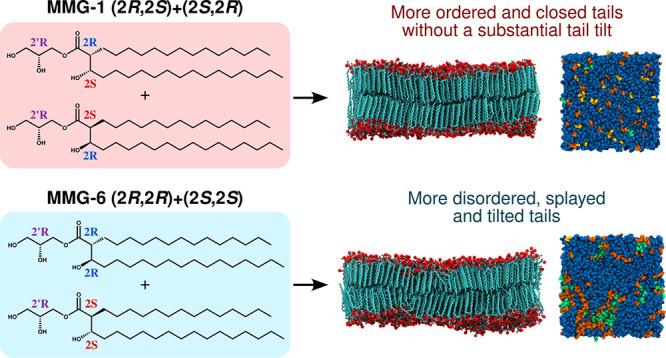

## Introduction

MMG is among the most immunostimulatory lipids found in the cell envelope of the vaccine Bacillus Calmette-Guérin (BCG).^1,2^ This potent bacterial lipid has been shown to induce strong activation of human dendritic cells, marked by the upregulation of maturation markers and the secretion of pro-inflammatory cytokines.^1^ The relatively simple structure of MMG comprises: (1) a glycerol moiety and (2) a mycolic acid esterified to one of the primary hydroxyl groups on the glycerol moiety (Figure 1a). Natural MMG lipids contain either an α- or a keto-mycolic acid, and both types of mycolic acids have comparable immunostimulatory properties.^2^

**Figure 1:**
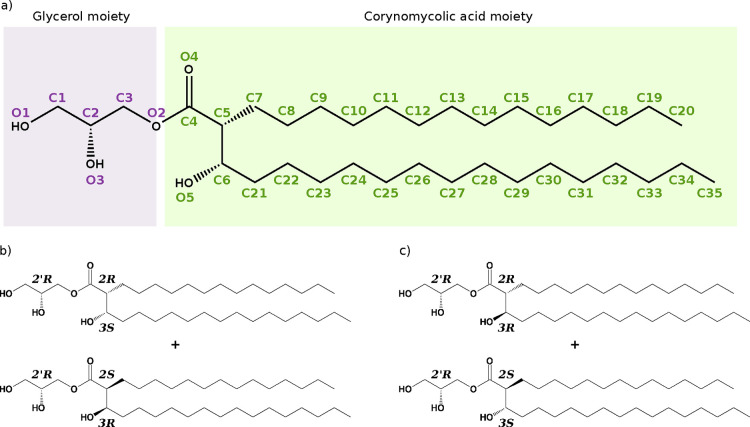
Molecular structures and atom naming of the synthetic MMG analogs simulated in this study. (a) Nomenclature and atom numbering. The names are illustrated with MMG-1 (2*R*,3*S*), and the same naming convention is used for all stereoisomers; (b) MMG-1 with (2*R*,3*S*)/(2*S*,3*R*) corynomycolic acid configurations, and (c) MMG-6 with native-like corynomycolic acid configurations (2*R*,3*R*)/(2*S*,3*S*).

Synthetic MMG analogs can be used for potentiating the immune response induced by subunit vaccines.^1^ Their lipid acid moiety is simplified and shorter in comparison to their natural counterparts, yet they possess comparable activity. Moreover, using a synthetic MMG analog as an alternative to naturally occurring MMG in vaccines has several benefits, including more simple and scalable synthesis, higher purity, and, in many cases, lower toxicity.^3^ Various MMG-analog-containing vaccine adjuvant formulations have been studied, including MMG/dimethyldioctadecylammonium (DDA) liposomes^2,3^ combined with Toll-like receptor (TLR) agonists,^4^ phytantriol/MMG hexosomes,^5^ and oil-in-water nanoemulsions.^6^ The most recent studies have focused on the further development of the cationic adjuvant formulation 09 (CAF09), which contains MMG, DDA, and the TLR-3 agonist poly­(I:C).^7–9^ The CAF09 formulation contains a specific *C*_32_ MMG analog, i.e., MMG-1, which displays a lipid acid stereochemistry that deviates from the native structure. The native structure corresponds to the (2*R*,3*R*) lipid acid stereochemistry, whereas MMG-1 is a 1:1 mixture of the (2*R*,3*S*)/(2*S*,3*R*) configurations (Figure 1b).^3^ In addition to a favorable immunostimulatory effect, MMG-1 has been shown to increase the colloidal stability of DDA liposomes, likely due to increased monolayer hydration and improved lipid packing properties.^3^

Interestingly, the lipid acid stereochemistry of different MMG analogs affects their phase behavior and the types of nanostructures the molecules self-assemble into at low temperatures.^10,11^ Analogs with a non-native lipid acid stereochemistry, e.g., MMG-1, assemble into a fully interdigitated subgel phase with a superlattice-like headgroup organization (*L*_c′_), while a native-like stereochemistry results in a noninterdigitated gel phase (*L*_β_).^11^ Generally, various MMG analogs have been shown to transition directly to the inverted hexagonal (*H*_II_) phase above the *T*_m_ before melting.^11^ The transition temperature, however, depends on the configuration and the chain length of the MMG analogs; native-like configurations have higher propensity for *H*_II_ formation at lower temperatures.^10,11^ The aforementioned differences appear to correlate with immunostimulatory properties in vitro: neat MMG-1 has been shown to induce more robust immunoactivation and to be less cytotoxic than an otherwise identical analog with a native-like lipid acid stereochemistry.^10^ In contrast, in vivo studies with an arrangement of DDA:MMG dispersions suggest that changing the MMG-1 lipid acid stereochemistry does not result in reduced immune response when the MMG analogs are incorporated into DDA liposomes.^12^

Here, we explored the physical mechanisms that allow MMG-1 to form interdigitated structures at low temperatures. Protrusion of lipid hydrocarbon chains into the opposing leaflet (i.e., interdigitation) is a complex phenomenon that, for other types of lipids, has been shown to occur either spontaneously or to be induced by external factors.^18^ These factors include changes in pressure or pH, as well as exposure to membrane-active proteins or certain small molecules.^18–17^ For MMG analogs with a lipid acid stereochemistry differing from their natural counterparts, self-assembly into interdigitated structures is a spontaneous process.^11^ Various structural features of lipids have previously been shown to correlate with their propensity for spontaneous interdigitation. For example, for phospholipids, spontaneous interdigitation is linked to factors that include chain asymmetry, headgroup structure, and positioning, as well as whether ester or ether linkages are present.^18,13–21^ While these studies have provided valuable insights into interdigitation specific to phospholipids, the mechanisms of MMG interdigitation may be different.

The aim of this study was to obtain insights into the molecular interactions, which cause differences in the interdigitation propensity and phase behavior of MMG analogs with alternative and native-like lipid acid configurations. Our hypothesis was that stereochemistry exerts a profound influence on the dynamics and spatial orientation of lipids, which affect the relative free energy of the possible different phases, eventually resulting in a specific phase being preferred. We used atomistic MD simulations to compare these properties between MMG-1 and another MMG analog, i.e., MMG-6, below their respective *T*_m_s. Only the lipid acid stereochemistry differs between these two analogs; their molecular structures are otherwise identical.^10^ Based on previous work, which highlights slow kinetics of interdigitation of lamellar bilayers,^22^ we concluded that simulating all stages of the self-assembly process from a random distribution of lipids to a fully equilibrated interdigitated membrane was not feasible for an all-atom system with the current computational resources, and the lipids could not be coarse-grained due to loss of stereochemistry. Therefore, noninterdigitated and interdigitated starting structures were constructed to investigate the conformations, dynamics, and interactions of the two MMG analogs of interest.

## Methods

### Lipids

Two synthetic MMG analogs were simulated: MMG-1 and MMG-6. Martin-Bertelsen et al. showed, using X-ray scattering, that MMG-1 forms an interdigitated *L*_c′_ phase, whereas MMG-6 assembles into a noninterdigitated L_β_ phase below their respective *T*_m_s in excess buffer.^11^ These two analogs display identical chain lengths (C_14_/C_15_) and glycerol stereochemistry (2*′R*). The atom nomenclature used throughout this article is listed in Figure 1a. Both MMG analogs consist of a racemic mixture of two corynomycolic acid variants. The MMG-1 mixture has a (2*R*,3*S*) and a (2*S*,3*R*) configuration of the lipid acid moiety (Figure 1b). The corresponding stereochemistries in MMG-6 are (2*R*,3*R*) and (2*S*,3*S*) (Figure 1c).

### Systems

The initial molecular structures of the MMG analogs were built using the Ligand Reader & Modeler utility of the CHARMM-GUI Input Generator.^23^ Both MMG-1 and MMG-6 were simulated in three different membrane configurations: (1) a noninterdigitated single bilayer, (2) a noninterdigitated double bilayer, and (3) a fully interdigitated double bilayer (Table 1). These configurations were chosen based on the X-ray scattering study by Martin-Bertelsen et al.,^11^ which showed that MMG molecules self-assemble into poorly hydrated lamellar structures at low temperatures. We used double bilayer configurations to represent these structures as they were described to contain little or no water. In addition, MMG-1 molecules have been observed to form the *L*_c′_ phase at our simulation temperatures, both in excess buffer and in anhydrous samples.^11^ A similar system setup has previously been used to model *stratum corneum* lipids, another poorly hydrated multilayer membrane structure.^24,25^ The single bilayer configuration was included to allow a comparison of different hydration states. All systems contained one of the two analogs as a 1:1 mixture of two stereoisomers.

**Table 1:** Summary of the Simulated Systems and Simulation Times^a^

system	MMG analog	# MMG molecules	# water	production run (ns)
single bilayer	MMG-1	1000	50,060	2500
	MMG-6	1000	49,357	2500
double bilayer	MMG-1	2000	50,132	2500
	MMG-6	2000	49,926	2500
interdigitated	MMG-1	2000	93,054	2500
	MMG-6	2000	89,175	2500

a The systems contained 1:1 mixtures of two stereoisomers.

System sizes were selected to allow for an accurate representation of possible interdigitation or membrane rippling in the noninterdigitated membranes. Finite-size effects are known to quench certain membrane phenomena, e.g., ripple phase formation^26,27^ and membrane undulations.^28^ Moreover, system size has been shown to affect the extent of stretch-induced interdigitation with united atom MD simulations.^29^ Therefore, all membranes were configured to contain 500 MMG molecules per leaflet, with two leaflets in the single bilayer system and four leaflets in the noninterdigitated and interdigitated double bilayer systems. The membranes were built with Packmol^30^ and solvated using *gmx solvate* after increasing the box size by 3 nm along the *z*-axis on both sides of the membrane. Any water molecules added to the hydrocarbon membrane core were subsequently removed.

### Molecular Dynamics Simulations

The lipids were modeled with the CHARMM General Force Field (CGenFF),^31^ with the CHARMM compatible TIP3P water model.^32^ The topologies and CGenFF parameters were obtained using the CHARMM-GUI ligand reader and modeler.^23^ Generally, the CGenFF is optimized to model small molecular drugs, and CHARMM36 ^33,100^ is used in lipid membrane simulations. Therefore, the atom types, partial charges, and dihedral potential functions were compared to the corresponding parameters found in the analogous lipid structures in CHARMM36. The CGenFF parameters were not changed because these parameters deviated only slightly from those produced by CHARMM36 and since the CGenFF penalty scores were 1 or below. Generally, parameters’ penalty scores lower than 10 indicate a reasonable analogy between the generated and existing parameters in the force field and do not require further validation.^34^

All MD simulations were performed using the GROMACS-2024.2 simulation package.^35^ The equations of motion were integrated by using a time step of 2 fs. The parallel linear constraint solver (P-LINCS) algorithm^36^ was used for constraining the bonds involving hydrogen atoms. The neighbor list cutoff for nonbonded interactions was set to 1.2 nm, as was the Lennard–Jones cutoff; the force was configured to switch off between 1.0 and 1.2 nm. The real-space Coulomb cutoff was set to 1.2 nm, and the particle-mesh Ewald (PME) method^37^ was used for computing the long-range electrostatic interactions using cubic interpolation and a maximum spacing of 0.12 nm for the fast Fourier transform grid.

The membranes were equilibrated with the following four-step scheme: (1) 50 ps with a 1 fs time step and using a canonical (NVT) ensemble at 348 K (75 °C) without position restraints, (2) 10 ns with a 2 fs time step continuing in NVT conditions at the same temperature, but now with restraining the lipids from their primary hydroxyl oxygen, O1 (see Figure 1a for atom numbering) with a harmonic force constant of 200 kJ mol^–1^ nm^–2^, (3) 10 ns with a 2 fs time step using a NPT ensemble with 1 bar pressure using the same temperature and position restraints as in the previous step, and 4) simulated annealing from 348 to 288 K over 60 ns (equating to a cooling rate of 1 K/ns) in NPT conditions without position constraints. The membranes were then allowed to equilibrate at the simulation temperature for 2 μs, and the production runs were considered to occur between 2 and 2.5 μs (Table 1). The simulation temperature was kept at 288 K, which is below the experimental *T*_m_ of both MMG-1 (332.15 K) and MMG-6 (291.15–295.15 K) under excess buffer conditions.^11^ To ensure the correct target temperature throughout the system, Langevin dynamics were applied. Semi-isotropic pressure coupling was applied along the *xy*- and the *z*-directions using the c-rescale barostat,^38^ with a compressibility of 4.5 × 10^–5^ and a 5.0 ps time constant to achieve a pressure of 1 bar. The center of mass translational velocity was removed for the whole system.

### Analyses

Potential energy, temperature, pressure, and area per lipid (APL) were monitored to ensure that equilibrium was reached. Based on the APL convergence, the final 500 ns of the simulations were used for all analyses (Figures S1 and S2). All analyses were conducted with the GROMACS-2024.1 and GROMACS-2024.2 analysis tools and custom Python codes using the MDAnalysis library.^39^ Visualizations were produced with VMD-1.9.4.^40^ Unless stated otherwise, the average values and standard deviations were obtained from the production run without block averaging.

#### Membrane Properties

The APL was calculated using two methods: (1) by multiplying the box dimensions in the *x*- and *y*-directions with each other and dividing the result by the number of lipids in one leaflet (APL_*xy*_) and (2) through 2D Voronoi tessellation^41,42^ (APL_vor_). Using the latter method allows the determination of individual APL values for each lipid, whereas the former gives only the average APL for the whole membrane with the assumption that the membrane is flat. The APL_vor_ was calculated only for the leaflets in contact with water; see the Supporting Information for a further description of the analysis. Membrane thickness values were obtained from the average membrane electron density profile peak values obtained using *gmx density*. The electron density profiles were calculated along the *z*-axis with the assumption that the membrane is planar and its surface is perpendicular to the *z*-axis. Thus, the membrane thickness describes the overall thickness of the whole membrane structure, with all lamellar layers considered. The thickness of the individual bilayers was also determined for the double bilayer configurations; the distance considered in this case extended from the outer leaflet peak to the electron density peak at the center of the membrane.

#### Tail Properties

Hydrocarbon tail order was investigated by analyzing the deuterium order parameters defined as

$$S_{\mathrm{CD}}=\frac{3}{2}\left\langle \cos^{2}\;\theta_{\mathrm{CD}}\right\rangle -\frac{1}{2}$$ 1

where θ_CD_ is the angle between the bilayer normal (i.e., the *z*-axis) and the carbon-deuterium vector, with the deuterium atom position predicted based on the coordinates of alkyl chain carbon atoms. The order parameters were calculated by averaging five 100 ns blocks, and the resulting block averages were subsequently used to derive the final average and standard deviation. The average angle between the tail vectors (C20–C7, C35–C21) and the bilayer normal was determined using *gmx gangle* (see Figure 1a for atom numbering). Given the planar nature of the membranes, the normal of the bilayer was assumed to be the *z*-axis. The angle between the headgroup and the tail vectors extending from the central carbon atom to the terminal atoms (C5–C20, C5–C35) was calculated in a similar way. Tail splay angles were calculated with *gmx gangle* by selecting (1) second tail carbon atoms C9 and C22 (splay 1) and (2) terminal carbon atoms C20 and C35 (splay 2). In both cases, the same central carbon atom, i.e., C5, was selected. In addition, we computed the tail rotational autocorrelation function (ACF_rot_) using a vector extending from the first carbon atom to the terminal carbon atoms (C7–C20, C21–C35). The equation for calculating the autocorrelation function (ACF) of a given property *f* is defined as

$$C_{f}\left(t\right)={\left\langle f\left(\tau \right)f\left(\tau +t\right)\right\rangle }_{\tau }$$ 2

where τ is the time origin, and *t* is the elapsed time. Tail-tail radial 2D distribution functions (RDFs) were determined in the *xy* plane to assess tail packing. The individual centers of masses (COMs) of the atom selection C7–C35 were used for the analysis to describe the tail positions.

#### Machine Learning (ML) Analysis of Molecular Conformations

To analyze MMG conformations, we used the ML-based technique of Davies et al.^27^ This involves a cluster analysis of the 2D distributions from the radial-angular, three-particle correlation function (*g*_3_).^43^ The atom triplet in *g*_3_ consists of the central atom (B), its nearest neighboring atom (A), and any atom within a predetermined cutoff distance (C). Essentially, the function expresses the probability of finding atom C at a distance *r*_BC_ from the central atom B when the vectors *r⃗*_BA_ and *r⃗*_BC_ form an angle θ_ABC_, see Figure 1 in Davies et al.^27^ Either intra- or intermolecular local structures can be analyzed, depending on atom selection.

We followed the protocol outlined in the original work for clustering similar *g*_3_ distributions together using unsupervised ML.^27^ The final 50 ns (501 frames) of the simulation were used for *g*_3_ calculations. All other carbon atoms of the lipid acid moieties, except for the carbonyl carbon C4, were chosen for independent analysis for each MMG molecule, thus giving the intramolecular structure for each lipid in isolation. The radial cutoff was set to 15 Å, with 201 radial bins and 101 angular bins. The similarities of the individual *g*_3_ distributions were compared using a structural similarity index metric to create a similarity matrix.^44^ The dimensionality of the 10,000 × 10,000 similarity matrix was reduced to 2D with t-distributed stochastic neighbor embedding (t-SNE).^45^ t-SNE was randomly initialized, and the perplexity and early exaggeration were set to 100 and 4, respectively. Hierarchical Density-Based Spatial Clustering of Applications with Noise (HDBSCAN)^46^ was used for clustering similar *g*_3_ distributions together from the 2D data with the minimum cluster size parameter set to 50. The results were validated by confirming the spatial separation of the clusters also with independent dimensionality reduction using principal component analysis (PCA). It was possible to validate the results in this fashion since the t-SNE was randomly initialized.

#### Analysis of the MMG Headgroup

For the analysis of the MMG hydrophilic moiety (i.e., the glycerol moiety or the glycerol moiety and the hydrophilic regions of the mycolic acid), direct MMG-MMG and MMG-water hydrogen bonding were analyzed by defining a hydrogen bond to exist, if the donor and acceptor were within a 3.5 Å distance and the acceptor–donor-hydrogen angle was ≤30°. The number of hydrogen bonds per molecule was calculated by dividing the number of hydrogen bonds by the number of MMG molecules in the system. The tail-corrected hydrogen bond ACFs (eq 2) were also computed to assess the stability of the hydrogen bonds between and within MMG molecules. For the 2D headgroup-headgroup RDF calculations, the individual hydrophilic moiety (C1–C6, O1–O5) COMs were used as both the reference and selection. To assess the dynamics at the lipid–water interface, the ACF_rot_ of the vector extending from the C1 to C3 atoms of the glycerol moiety was determined (atom numbering in Figure 1a); eq 2 was used.

## Results

All systems maintained mostly planar configurations within the simulation time (Figure 2). The visualizations showed areas with disordered lipid tails for the single- and double bilayer MMG-6 systems, which indicates that the systems display properties resembling those of the ripple phase. Based on the visual observation alone, the alkyl chains are aligned parallel to the lipid bilayer normal in the interdigitated systems, and this type of tail packing also remains in the noninterdigitated systems containing MMG-1. Some degree of partial interdigitation with accompanying membrane deformations was observed for all systems with noninterdigitated starting configurations (Figures 2a,b and S5a,b).

**Figure 2:**
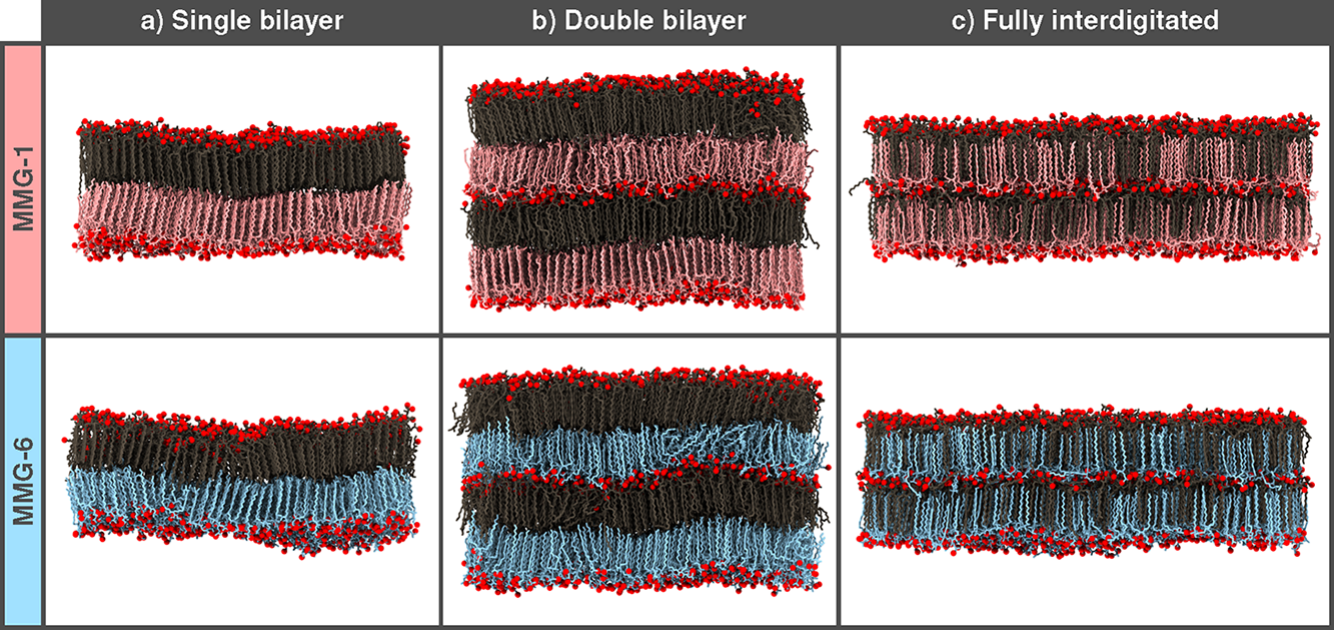
Snapshots from the final frames of the simulations (a) single bilayer, (b) double bilayer, and (c) fully interdigitated systems. The leaflets are colored either pink and dark taupe (MMG-1) or blue and dark taupe (MMG-6) to enhance the visibility of possible tail interdigitation. Water and hydrogen atoms on the MMG molecules are omitted for clarity. The O1 oxygen atoms in the primary hydroxyl groups on the glycerol moieties are displayed as red spheres. See Figure 1a for atom numbering.

### Membrane Properties

The membrane dimensions were investigated through APL and membrane thickness estimations. The APL was derived either from the box size in *x*- and *y*-directions (APL_*xy*_) or through 2D Voronoi tessellation (APL_vor_). Both APL_*xy*_ and APL_vor_ results indicate that the MMG-6 molecules tend to occupy larger areas in the noninterdigitated systems (Table 2, Figure 3). Moreover, the effect of interdigitation on the membrane area is clearly demonstrated by the APL_*xy*_ results: the lipids in the interdigitated double bilayer systems take up approximately 1.7 (MMG-1) to 1.6 (MMG-6) times larger areas in comparison to the corresponding noninterdigitated double bilayer (Table 2). Interestingly, the APL_vor_ distributions show a wider range of APL values for individual lipids in the interdigitated systems (Figure 3). Both APL_*xy*_ and APL_vor_ versus time plots demonstrate that the systems have reached equilibrium (Figures S1 and S2). Electron density-based membrane thickness analyses confirmed visual observations from the membranes; the MMG-6 molecules tend to form thinner membranes, with approximately 6 and 20 Å differences in comparison to MMG-1 with noninterdigitated single bilayers and double bilayers, respectively (Table 2). This difference in membrane thickness may originate partly from different tail and headgroup tilts. The average electron densities over the final 500 ns are presented in Figure S3.

**Table 2:** Membrane Properties of the Bilayer Structures Containing either MMG-1 or MMG-6^a^

	APL*_xy_* (Å^2^)		membrane thickness (Å)		bilayer thickness (Å)	
	Avg	StDev	Avg	StDev	Avg	StDev
single bilayer						
MMG-1	39.8	0.1	36.3	0.5	36.3	0.5
MMG-6	42.9	0.2	30.5	1.1	30.5	1.1
double bilayer						
MMG-1	41.2	0.1	83.2	0.9	41.6	3.7
MMG-6	45.7	0.1	63.7	2.4	31.9	2.3
interdigitated						
MMG-1	72.0	0.1	47.7	0.1	23.9	0.7
MMG-6	73.2	0.1	46.1	0.5	23.1	0.7

a APL_*xy*_ is the area per lipid. Membrane and bilayer thickness values are averages of five electron density peak-to-peak block average distances.

**Figure 3:**
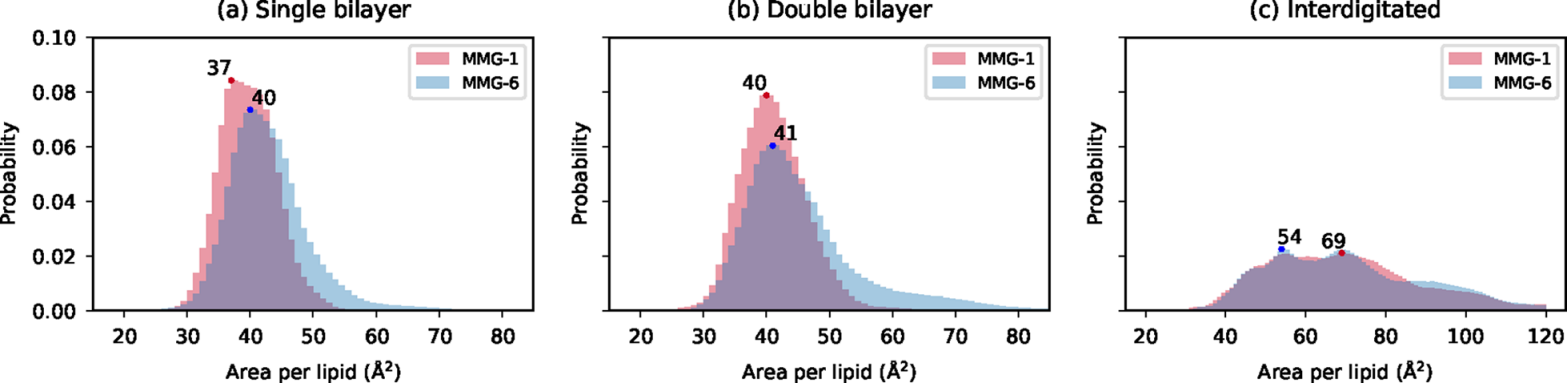
Probabilities of the area per lipid (APL_vor_) values were calculated through 2D Voronoi tessellation. The maxima are denoted with a circle with the corresponding value. The MMG-1 vs MMG-6 average values are 39.8 Å^2^ vs 43.0 Å^2^ (single bilayer), 41.2 Å^2^ vs 45.7 Å^2^ (double bilayer), and 72.1 Å^2^ vs 73.2 Å^2^ (interdigitated). The corresponding median values are 39.6 Å^2^ vs 42.2 Å^2^ (single bilayer), 40.8 Å^2^ vs 43.5 Å^2^ (double bilayer), and 68.7 Å^2^ vs 69.2 Å^2^ (interdigitated).

Only MMG-1 has been reported to assemble into fully interdigitated membranes below 25 °C, whereas MMG-6 has been proposed to form noninterdigitated structures at the temperature used for the simulations.^11^ In contrast to these experimental results, no apparent difference was observed from the mass density profiles (Figure S4). Both visualizations and the number density profile histograms show that the tails of MMG-1 and MMG-6 extend into the opposing leaflet, forming partially interdigitated regions (Figures S5 and S6). The degree of partial interdigitation is slightly greater for MMG-6, which is evident from the larger leaflet overlap. However, partial interdigitation is a phenomenon encountered with lipids both in the gel and the disordered phases and does not necessarily predict the formation of a fully interdigitated phase.^47,48^

### MMG Analogs Display Differences in Tail Order, Tilt Angles, and Conformations

Our results from the deuterium order parameter analysis (eq 1) show that the hydrocarbon chain order differs between noninterdigitated and interdigitated membrane structures as well as between MMG-1 and MMG-6 in the noninterdigitated systems. The interdigitated systems display comparable order parameter values for both MMG analogs (Figure 4c,f). These order parameters measured from the interdigitated membranes are higher than in the noninterdigitated systems, matching with the previous observation in which glycerol-induced interdigitated dipalmitoylphosphatidylcholine (DPPC) bilayers were observed to display a higher extent of acyl chain ordering in comparison to that of the noninterdigitated phase.^49^ The noninterdigitated systems have notable differences in the order parameters between the analogs; in particular, the MMG-6 tails are more fluid in these systems (Figure 4a,b,d,e). The tail vector ACF_rot_ (eq 2) was computed for each system to investigate the tail dynamics further (Figure S7). The ACF_rot_ data show overall faster tail dynamics for MMG-6, especially for tail 2, which further supports the order parameter results.

**Figure 4:**
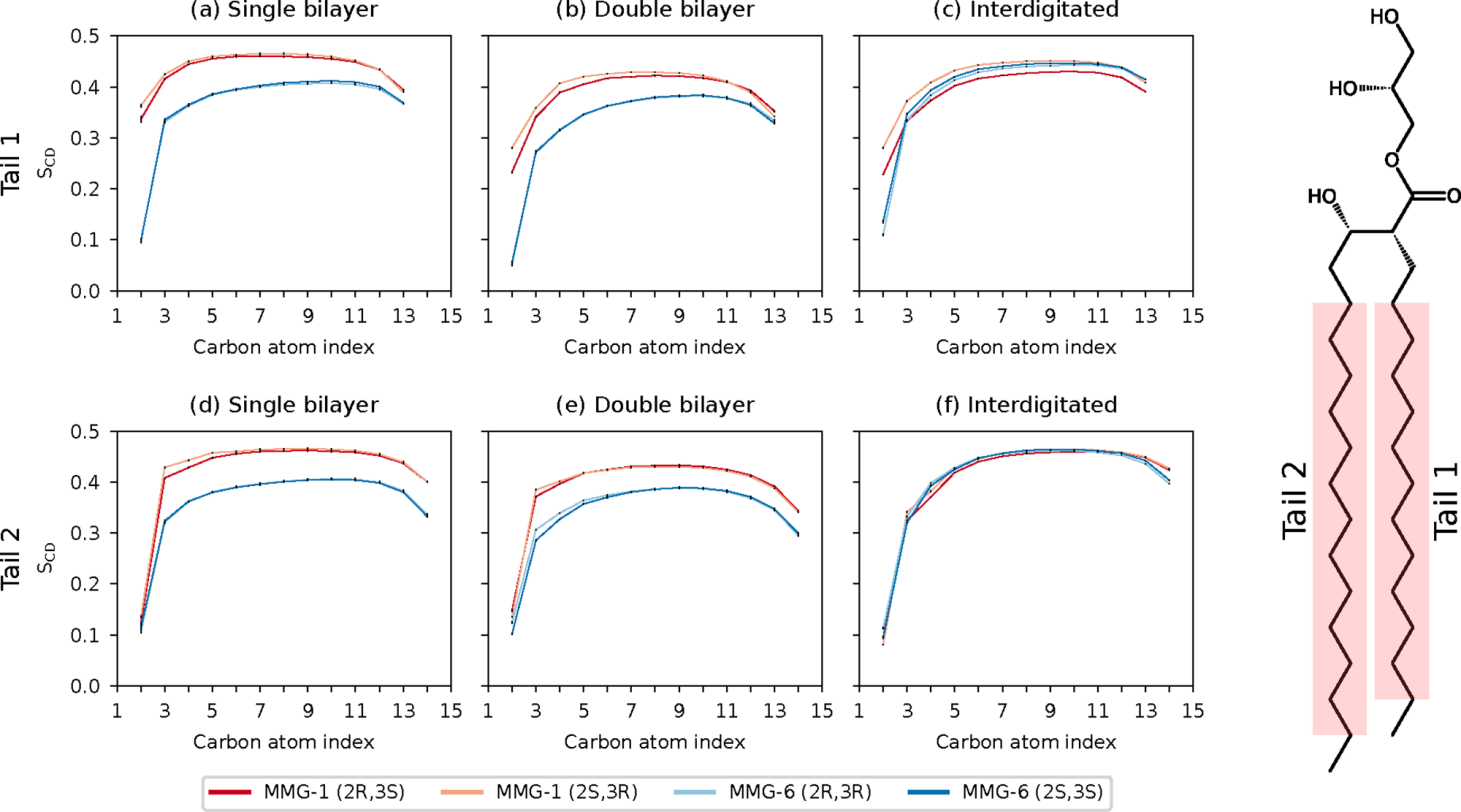
Deuterium order parameters (*S*_CD_) of hydrocarbon tails of the MMG analogs, see eq 1. Error bars and average values were obtained using block analysis with five 100 ns blocks. The molecular structure shows the atoms considered in the plot.

The tilt angle distributions measured from the final 500 ns of the simulation show that the MMG-6 tails adopt a more tilted position in the noninterdigitated cases with the maximum probabilities found in the range of 10.5° to 15° (tail 1) and 10° to 13.5° (tail 2) (Figure 5). The tail tilt angles of MMG-1 display larger similarity in the noninterdigitated systems to those in the interdigitated structures, with maximum peaks at angles found at 7.0° to 9.5° (tail 1) and 7.5° to 9.5° (tail 2). All stereoisomers have similar tail tilt angles in the interdigitated systems with maximum probabilities at 5.0° to 5.5° (tail 1) and 6.0° to 7.5° (tail 1). The difference in tilt angles between the two MMG analogs is most pronounced in the single bilayer structure (Figure 5a,d). Average tail-to-headgroup tilt angles showed a trend of larger angles for MMG-1 in comparison to the case for MMG-6, suggesting a less tilted orientation of the headgroup relative to the hydrocarbon tails (Figure S8). However, these angles were widely distributed in all cases.

**Figure 5:**
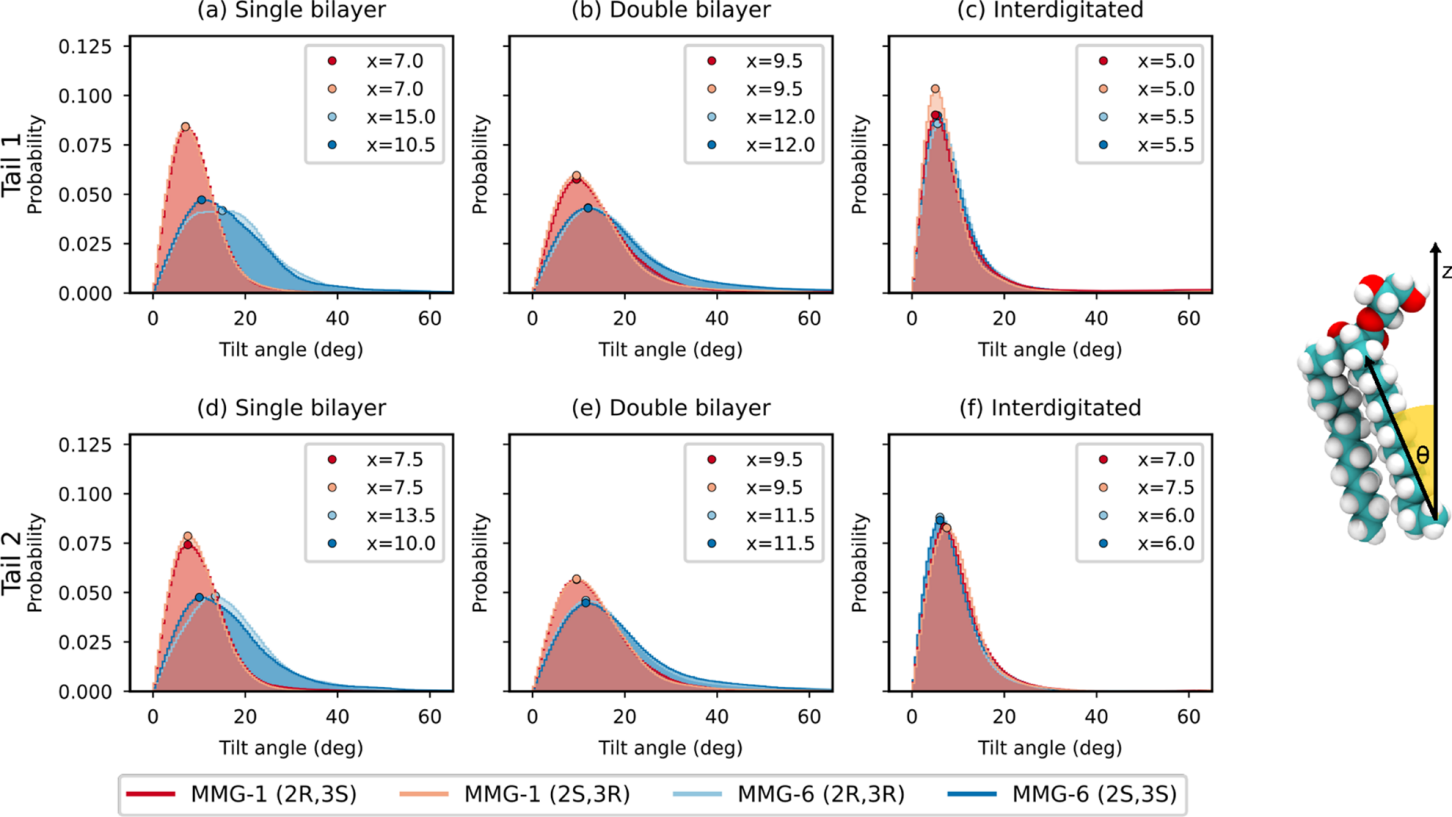
Angle between the tail vector extending from the terminal carbon to the first carbon in the tail and the bilayer normal. The angles at maximum probabilities have been marked with circles with distinct colors for each stereoisomer; the corresponding angle annotations are shown in the inset.

We analyzed two splay angles: the C9–C5–C22 splay angle close to the lipid acid moiety (splay 1) and the C20–C5–C35 splay angle, which considers the full length of the tails (splay 2) (Figure 6). Both MMG-1 and MMG-6 molecules show similar distributions of splay angles with two distinct peaks of splay 1. With splay 1, for all the cases, the majority of the lipids have the upper part of their tails forming an angle close to 95° and a second angle close to 120° (Figure 6a–c). With MMG-6, the probability distribution is more evenly spread between these two peaks. This indicates a higher probability for a larger splay near the area connecting glycerol and the lipid acid moiety. A larger splay angle means that these lipids are more likely to take up a larger volume within the membrane. Our APL_*xy*_ and APL_vor_ results support this idea. The splay 1 distributions also show a small population of angles around 150°, which is visible mainly in the interdigitated membrane configuration. The probabilities of splay angle 2 show higher similarity between the systems, with the majority of the splay angles being close to 15° (Figure 6d–f). This angle corresponds to a closed-tail conformation. A second, more subtle, peak can be seen at approximately 27°. Both splay 1 and splay 2 show a shift in the ratio of the peaks in the interdigitated system for MMG-1 with the second peak being more prominent than in other systems. This can be explained by the results from the clustering analysis presented in the next section (Figure 7). A similar shift is seen between the single/double/interdigitated systems. The positions of the peaks are constant; just the ratio of the components differs. A similar trend was not observed in the case of MMG-6.

**Figure 6:**
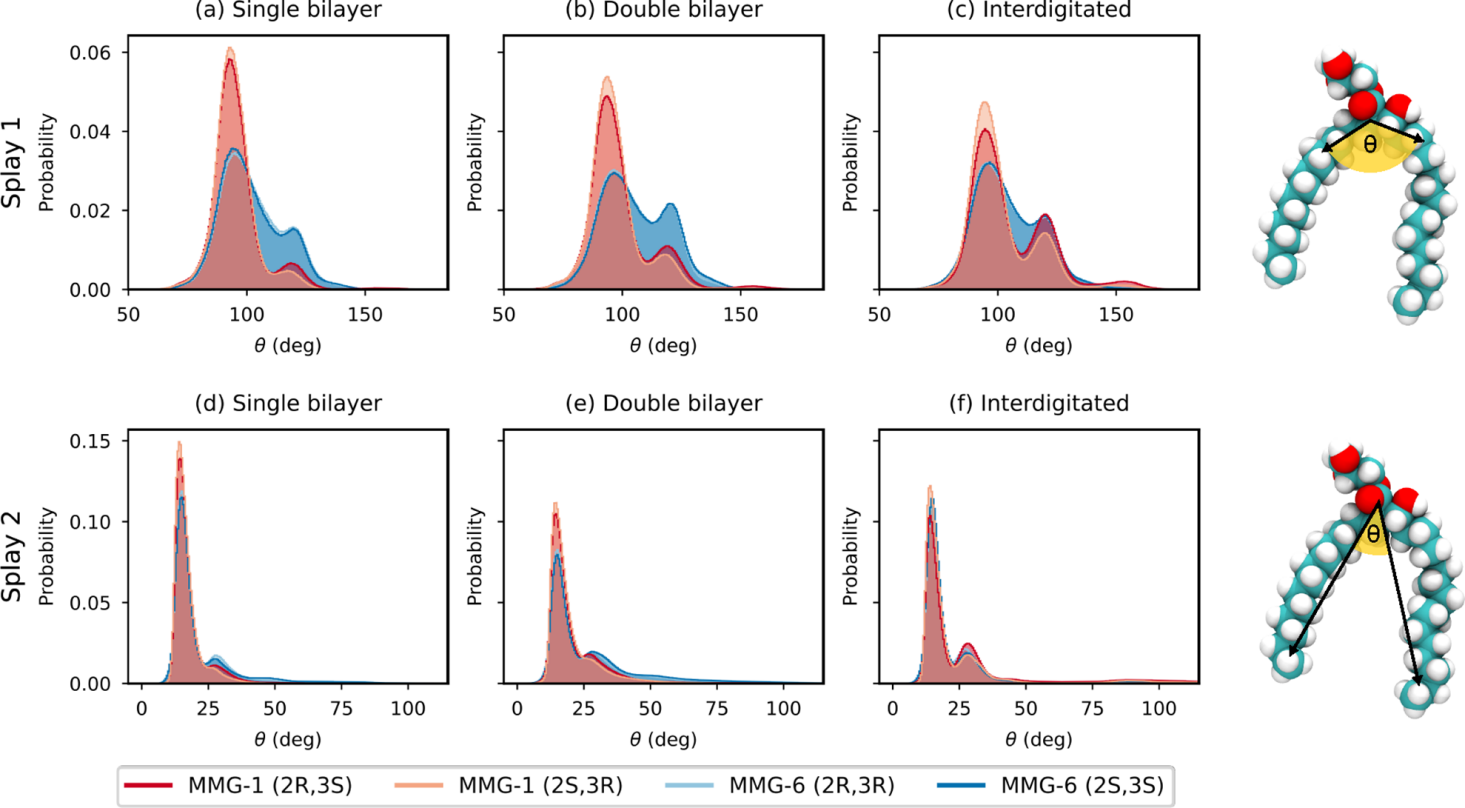
Splay angles between the tail carbon atoms and the central carbon atom of the corynomycolic acid. Splay 1 (a–c) depicts the angle formed by the central carbon atom and carbon atoms near the lipid acid moiety. Splay 2 (d–f) considers the central carbon atom and the terminal carbon atoms on each tail.

**Figure 7:**
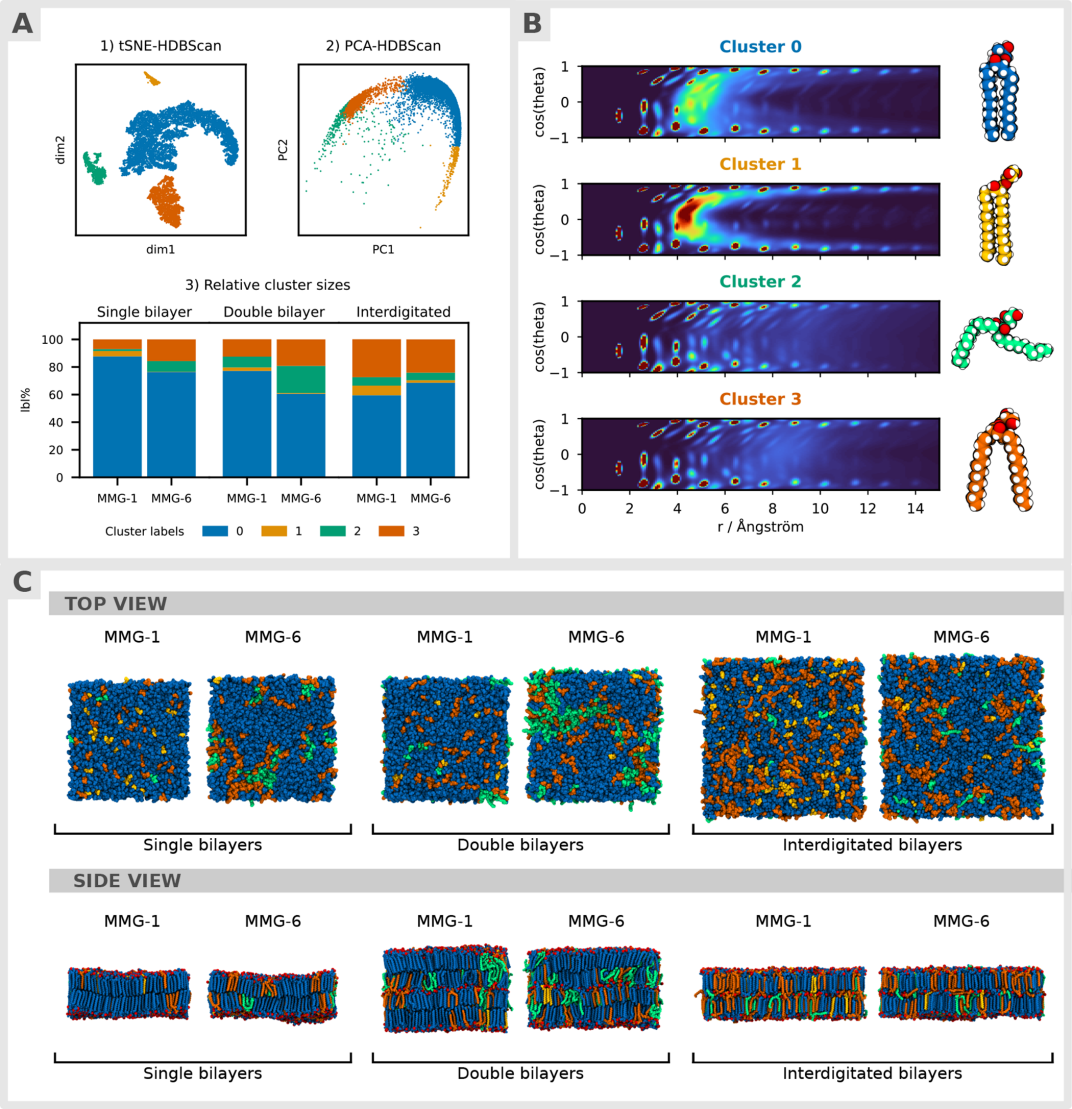
Clustering and visualizations of MMG analogs based on the radial-angular, three-particle correlation function (*g*_3_) distributions of the carbon atoms in their hydrocarbon tails. (A1) 2D t-SNE embedding of the data and clustering with HDBSCAN, (A2) 2D PCA embedding with the same sample point coloring, (A3) Distributions of label percentages in each system. (B) Average *g*_3_ distributions of each tail conformation type with corresponding visualization. Blue = (0) closed tails, less ordered; orange = (1) closed tails, more ordered; green = (2) splayed, at least one tail disordered; red = (3) splayed, both tails ordered (C) Visualization of membranes at the final time frame with MMG molecules colored according to their lipid type. The oxygen atom in the primary hydroxyl group on the glycerol moiety is colored red in the side view.

### Clustering Analysis

Clustering of the *g*_3_ distributions revealed four distinct lipid type populations rather than a single homogeneous mixture (Figure 7). Visualizations of the *g*_3_ distributions are described in detail with annotations in Figure S9. The peaks near cosθ = 1 correspond to atoms *n* + 1 bonds away from the central atom on the same side of the nearest-neighbor atom in the lipid tail hydrocarbon chain.^27^ Similarly, the peaks near cosθ = −1 are equivalent atoms *n* bonds away on the opposite side of the nearest neighbor. While the first peaks are always singular, the second peak and onward can be split into trans and gauche peaks. High atom order in stiff tails produces more pronounced and higher trans peaks, whereas fluid tails appear as more diffuse peaks and higher gauche peak density.^27^ Increased density near the center of the plot (peaking around the radial distance 4 < *r* < 6 Å) indicates atoms on the other tail and corresponds to increased tail packing in that specific atom location. Based on the *g*_3_ distributions and visualizations, the clusters can be categorized into four different lipid structural motifs as follows:

- 0 = closed tails, ordered;
- 1 = tightly closed tails, ordered;
- 2 = splayed, at least one tail disordered;
- 3 = splayed, both tails ordered.

Interestingly, a similar set of four clusters was also found in a study of the main phase transition of pure DPPC membranes.^27^ Together, these results suggest the universality of the above four structural motifs.

Visualizations of the *g*_3_ distributions and individual lipids show clear differences in intramolecular tail packing (Figure 7b). The hydrocarbon tails of clusters 0 and 1 lipids are more densely packed, while clusters 2 and 3 display more diffuse peaks at larger radial values, indicating splayed tails. Overall, cluster 1 shows the tightest intramolecular tail packing. The trans/gauche peak ratios are, however, within a similar range for clusters 0, 1, and 3 (Figure S9). Splayed lipid types 2 and 3 clearly differ in their trans/gauche peak ratios; cluster 2 lipids have the lowest trans/gauche peak ratio and are thus the most disordered lipid type. Distinct tail ACF_rot_ curves (eq 2) and splay angle distributions confirm the proposed classification (Figures S10 and S11). All four lipid types (Figure 7b) were found for both MMG-1 and MMG-6 (Figure 7a,c). The populations of tightly packed type 1 lipids are larger for MMG-1, whereas MMG-6 displays a higher number of more disordered type 2 lipid structural motifs (Figure 7a). Interestingly, the number of type 3 lipids is clearly higher in the interdigitated systems in comparison to the other systems in the case of MMG-1, matching the shift in the splay angle peak ratios (Figure 6). Moreover, the ratios of the type 0 lipids are reversed for MMG-1 and MMG-6 in the interdigitated systems in comparison to other systems. To improve the understanding of the tail packing and interactions, we analyzed tail–tail RDFs. Unexpectedly, none of the systems displayed a high order of intermolecular tail packing, and no evident difference was found between MMG-1 and MMG-6 in tail interactions (Figure S12).

### Hydrophilic Moiety Interactions and Dynamics

Headgroup interactions have been suggested to have a crucial impact on the ability of certain lipids to form interdigitated structures below *T*_m_.^50^ This was explored by analyzing hydrogen bonding, hydration, and dynamics of the MMG hydrophilic region, which was considered here to consist of the glycerol moiety and the hydrophilic part of the lipid acid moiety. Hydrogen bonding analysis revealed no clear differences between MMG-1 and MMG-6 in intermolecular hydrogen bonding (Table 3). The hydrogen bond analysis shows, nevertheless, that MMG-1 is more inclined to form intramolecular hydrogen bonds both in single and double bilayers. However, it should be noted that the number of intramolecular hydrogen bonds is generally very low in all cases. To further elucidate hydrogen bonding behavior in our systems, we computed the hydrogen bond ACFs from overall direct hydrogen bonding (intra- and intermolecular combined); the results for this analysis can be found in Figure S13. Although the tail-corrected hydrogen bond ACFs are almost indistinguishable between MMG-1 and MMG-6, the decay profiles display differences between the systems. The initial decay of the hydrogen bond ACFs is overall the fastest with the single bilayer system, indicating that the double bilayer configurations have more stable hydrogen bonds.

**Table 3:** Number of the Intermolecular and Intramolecular Hydrogen Bonds^a^

	intermolecular H-bonds		intramolecular H-bonds	
	Avg	StDev	Avg	StDev
single bilayer				
MMG-1	1.81	0.05	0.16	0.02
MMG-6	1.84	0.05	0.09	0.01
double bilayer				
MMG-1	3.03	0.03	0.20	0.01
MMG-6	3.07	0.03	0.14	0.01
interdigitated				
MMG-1	2.47	0.03	0.17	0.01
MMG-6	2.55	0.03	0.15	0.01

a The number of hydrogen bonds is presented as average values per molecule with standard deviation.

Contrary to the other membrane configurations studied here, all MMG molecules are in contact with water in the single bilayer system. With order parameters and the tail tilt showing the most considerable differences in the single bilayer system (Figures 4 and 5), we investigated if these properties could be linked to differences in headgroup hydration. Thus, we analyzed the number of hydrogen bonds formed by individual acceptor and donor groups and water molecules (Table 4). The noninterdigitated single and double bilayer configurations showed clear differences in this regard, with MMG-6 forming more hydrogen bonds with water through its O4 and O5–H groups (Table 4). These atom groups are located in the lipid acid moiety, which displays a different 3D configuration than the lipid acid moiety of MMG-1.

**Table 4:** Number of Hydrogen Bonds with Water Formed by Selected Atom Groups^a^

	O1–H		O2		O3–H		O4		O5–H	
	Avg	SD	Avg	SD	Avg	SD	Avg	SD	Avg	SD
single bilayer										
MMG-1	1.29	0.03	0.05	0.01	1.34	0.03	0.40	0.02	0.75	0.02
MMG-6	1.32	0.03	0.04	0.01	1.32	0.03	0.52	0.02	0.84	0.03
double bilayer										
MMG-1	0.66	0.02	0.03	0.01	0.68	0.02	0.22	0.01	0.39	0.02
MMG-6	0.68	0.02	0.02	0.01	0.69	0.02	0.29	0.01	0.45	0.02
interdigitated										
MMG-1	0.76	0.02	0.03	0.01	0.78	0.01	0.38	0.01	0.60	0.02
MMG-6	0.74	0.02	0.03	0.01	0.75	0.02	0.42	0.01	0.62	0.02

a The number of hydrogen bonds is presented as an average value per molecule with a standard deviation.

The RDF results show that the hydrophilic moiety COMs are, on average, at a similar distance from each other, and no clear difference in this regard was found between the MMG analogs (Figure 8). As expected, the interdigitated systems have the main RDF peak at a greater distance in comparison to the case of the noninterdigitated systems. None of the systems exhibit clear headgroup order, contrary to experimental results indicating molecular superlattices with MMG-1 headgroups.^11^ This result could be, for instance, due to the system being kinetically trapped and at a metastable state.

**Figure 8:**
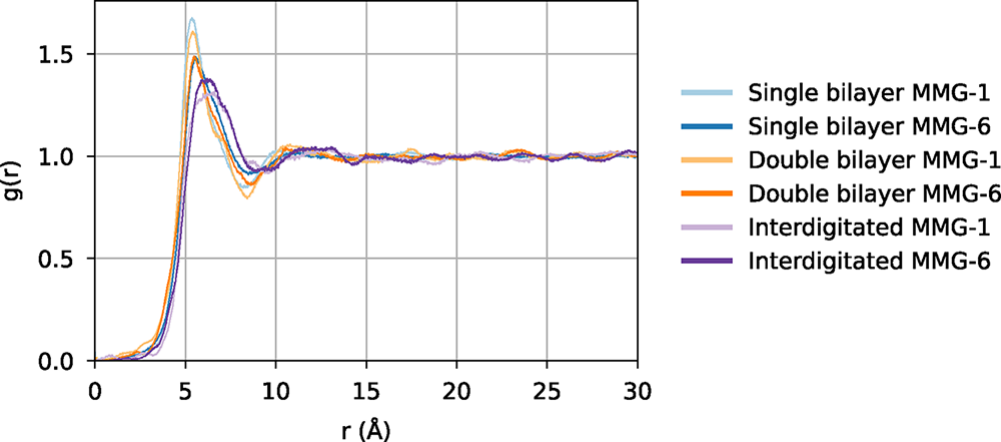
Average 2D radial distribution function *g*(*r*) where the distribution is a function of the distance between the COMs of hydrophilic MMG moieties. Only outer leaflet lipids are considered in the analysis; the radial distribution functions have been calculated separately for each leaflet and then averaged.

The glycerol moiety vector’s ACF_rot_s deviate slightly between the MMG stereoisomers. A sum of two exponential functions was found to fit all decay curves,

$$a_{1}e^{-t/\tau_{1}}+a_{2}e^{-t/\tau_{2}}+b$$ 3

where *a*_*i*_s are the amplitudes, *b* defines the function offset, *t* is time, and τ_*i*_s are the characteristic rotation times. The characteristic times are given in Table 5.

**Table 5:** Characteristic Rotation Times and Amplitudes Derived from Curve Fitting to Rotational ACFs Using Two Exponential Functions^a^

system	stereoisomer	τ_1_ (ns)	*a*_1_	τ_2_ (ns)	*a*_2_
single bilayer	MMG-1 (2*R*,3*S*)	2.1	0.23	57.3	0.12
	MMG-1 (2*S*,3*R*)	2.0	0.22	45.1	0.12
	MMG-6 (2*R*,3*R*)	1.6	0.22	77.0	0.10
	MMG-6 (2*S*,3*S*)	1.6	0.21	86.0	0.10
double bilayer	MMG-1 (2*R*,3*S*)	3.9	0.13	104.9	0.11
	MMG-1 (2*S*,3*R*)	3.5	0.12	90.9	0.10
	MMG-6 (2*R*,3*R*)	2.9	0.11	115.6	0.12
	MMG-6 (2*S*,3*S*)	2.9	0.11	124.9	0.12
interdigitated bilayer	MMG-1 (2*R*,3*S*)	6.6	0.13	104.4	0.13
	MMG-1 (2*S*,3*R*)	5.5	0.13	91.3	0.12
	MMG-6 (2*R*,3*R*)	3.8	0.09	132.1	0.14
	MMG-6 (2*S*,3*S*)	3.8	0.09	129.0	0.13

a τ_1_ = fast rotations, τ_2_ = slow rotations, and *a*_*i*_ = corresponding amplitude.

The characteristic times indicate the existence of two main rotational modes (fast and slow) for the glycerol moieties. Based on the amplitudes, the short time scale rotations contribute more to the overall ACF_rot_ in the single bilayer systems, whereas other systems display similar amplitudes between both rotational modes (Table 5). The MMG-1 glycerol moieties display a trend of slower short time scale rotations and faster long time scale rotations (Table 5). However, when both short and long time scale rotations are considered, the overall rotations of MMG-1 headgroups are likely faster in comparison to those of MMG-6. The differences between MMG-1 and MMG-6 in this regard are less pronounced in the noninterdigitated double bilayer systems. No clear indication of distinct ACF_rot_ could be observed for different clusters based on *g*_3_ analysis (Figure S14). These results imply that the headgroup rotational dynamics are unrelated to the tail conformations or packing; it is probable that this is more closely related to the extent of headgroup hydration.

## Discussion

Lipid interdigitation is a complex phenomenon originating from the interplay between minimizing tail packing energy and the number of gauche rotamers, while simultaneously reducing voids in the hydrophobic region.^18^ Our MD simulations show that lipid acid stereochemistry affects the tail fluidity, conformation, and tilt of synthetic MMG analogs. According to our results, the MMG-1 tails are, in general, found to have a higher extent of ordering in noninterdigitated systems. The MMG-1 deuterium order parameters are relatively similar for all systems, whereas MMG-6 showed an apparent discrepancy between the order parameters in noninterdigitated and interdigitated states. It is possible that the propensity for interdigitation is related to a change in the entropic penalties for tail ordering.

Our results also show that lipid acid stereochemistry affects the tail tilt of MMG molecules: MMG-1 tails display a more upright orientation parallel to the bilayer normal compared to MMG-6 in noninterdigitated systems. Previous MD simulation work with emollients and disaturated phosphatidylcholines (PCs) in the gel phase has exemplified the link between molecular geometry and the overall membrane structure and properties such as tail tilts.^51^ For the case of emollients and disaturated PCs, the headgroup size or branching of the tails can result in energetically unfavorable distances between the molecules, and an increased tail tilt is required to achieve optimal tail packing. Similarly, the larger tail tilt of MMG-6 possibly serves to maintain an optimal tail packing distance, despite the larger tail splay near the glycerol moiety.^18,51^ Interestingly, tilt angles have been suggested to increase only up to a certain threshold until the bilayer interdigitates, at least in the case of alcohol-induced interdigitation of phospholipids;^52^ coarse-grained simulations of Kranenburg et al., did not, however, show an increase in the tilt angle,^53^ but this is likely due to the relatively large degree of coarse-graining. Therefore, it is possible that MMG-6 interdigitates when the tail tilt alone is not sufficient to maximize the van der Waals interactions. However, the enthalpic penalty of changing from a tilted tail configuration to that observed in the interdigitated state (Figure 5) may counteract this.

Interactions and crowding in the hydrophilic region have also been suggested to affect the propensity for interdigitation. In the case of phospholipids, substituting PC ester linkages with ether linkages allows the lipids to form interdigitated structures at atmospheric pressure.^15,21^ The tendency of ether-linked PCs for interdigitation has been attributed to decreased attraction in the hydrophilic region, i.e., through a reduction in the extent of hydrogen bonding through water molecules.^15^ The headgroup size has also been shown to play a role for interdigitation; bulkier headgroups have been shown to correlate with interdigitation in the case of PC versus phosphatidylethanolamine (PE),^15,50^ possibly due to increased headgroup repulsion and crowding, as well as the formation of voids in the hydrophobic region. While the MMG molecules did not display clear differences in intermolecular hydrogen bonding, MMG-1 was seen to have a slightly greater extent of intramolecular hydrogen bonding. This might relate to certain geometric configurations involving the headgroup and the hydroxyl group in corynomycolic acid. It should be noted, however, that MMG-1 and MMG-6 contain identical glycerol moieties, and the glycerol moiety itself has not been shown to affect the type of structure that MMG molecules self-assemble into.^10^

It is well-known that structural factors affect hydration-induced amphiphile self-assembly through differences in molecular shape and the subsequent intrinsic curvature of the molecules.^54^ The effect of stereochemistry on the characteristics of the self-assembled structures has been demonstrated experimentally with gemini surfactants^55–57^ and sphingomyelin.^58^ In the present study, the MMG-6 stereoisomers adopted more splayed and disordered tail configurations with a higher APL. It has been proposed that the native-like lipid acid configuration allows MMG-6 hydrocarbon chains to take conformations with larger spatial volumes in comparison to those of MMG-1.^11^ Both wide and small-angle X-ray scattering studies have previously shown that MMG-6 forms an inverted hexagonal (*H*_II_) phase at room temperature, whereas MMG-1 maintains planar structures at temperatures of up to 60 °C.^10,11^ As the *H*_II_ phase arises from the relatively small headgroup to tail ratio and subsequent spontaneous negative curvature induced for the monolayer,^59^ our results support the idea of a larger spatial volume occupied by the MMG-6 tails. Furthermore, previous Langmuir monolayer studies have indicated that a higher surface pressure is required for MMG-6 to transition from the liquid-expanded to the liquid-condensed phase compared to the case for MMG-1.^10^ This requirement for a higher surface pressure was attributed to the more wedge-shaped molecular structure of MMG-6, which restricts the formation of similar favorable hydrophobic interactions observed for MMG-1. Our simulation results align with this perspective, as evidenced by consistent observations in the order parameters, splay angle, and *g*_3_ clustering; all of these analyses indicate that the alkyl chains of MMG-6 lipids display higher alkyl chain entropies, which effectively increase the volume adopted by the alkyl chains and possibly hinder alkyl chain packing.

Although our simulation results show clear differences in the behavior and dynamics of the selected MMG analogs, our methodology and models possess also certain limitations. The MD simulation technique with all-atom resolution can investigate time scales of the order of a few microseconds. To overcome the inherently short time scale of all-atom MD simulations, we built planar starting structures already either in interdigitated or noninterdigitated state. However, even with readily built structures, the simulation time scales currently accessible with supercomputers may not be sufficient for observing full interdigitation due to possibly existing kinetic factors.^19,22^ With regular lipids, the use of a multiscale approach similar to that utilized by Shamaprasad et al. to study *stratum corneum* lipids^25^ could be a solution to obtain self-assembled structures below *T*_m_. However, this approach is impossible with MMG molecules as a result of a loss of stereochemistry during coarse-graining. In addition, achieving a gel phase is particularly difficult with the CHARMM36 force field without a considerable finite-size effect, and the phase behavior of lipid membranes at low temperatures has been shown to depend on the thermal history and the conditions they are prepared under.^26^ These limitations underline the need for further experimental validation to confirm the results on tail order parameters and tilting. Since membrane interdigitation involves a complex balance of forces, it should be acknowledged that other factors may also influence the interdigitation of MMG analogs. Nevertheless, the current study provides insight into the mechanisms that may explain the link between MMG stereochemistry and their tendency for interdigitation because most of the differences between the MMG analogs persist through different membrane configurations. Furthermore, the clustering analysis used in the study underlines how differences in overall membrane properties do not necessarily directly reflect how the individual lipids behave; the differences may arise from different proportions of certain lipid behavior.

The applicability of MD simulations for obtaining mechanistic insight, which helps guide the rational design of liposomal drug delivery systems, has been demonstrated in multiple studies;^60–63^ a similar approach can also be applied to the development of liposomal adjuvants. Understanding the relationships among the structure, dynamics, and interactions of a single component is essential for the development and optimization of more complex formulations. While neat MMG analogs are not used as standalone adjuvants, the simplistic one-component simulation approach of the current study can, however, be seen to pave the way for more complex simulation setups involving other formulation components. Even though neat MMG-1 has been shown to mediate higher immunoactivation than MMG-6 in vitro, studies with MMG/DDA liposomes have indicated similar immunoactivity between the analogs in vivo.^10,12^ This discrepancy in the results between neat analogs and formulations illustrates the importance of future simulations with all formulation components to further elucidate the biophysics of synthetic MMG analogs.

## Conclusions

In this study, we used MD simulations of various membrane configurations to investigate how the stereochemistry of the lipid acid moiety affects the molecular shape, dynamics, and interactions of MMG analogs. Our aim was to gain insight into the molecular interactions explaining why MMG-1 has been observed to self-assemble into the interdigitated subgel phase and MMG-6 into the noninterdigitated gel phase at low temperatures. The results revealed differences in the tail conformations and fluidity of the MMG lipids in the bilayers. Our findings on tail order parameters indicate that the interdigitation of MMG-1 could result from a change in the balance between entropy and enthalpy. However, further experimental validation is required to confirm these results. The results also support the previously suggested idea of MMG-6 being more prone to form an inverted hexagonal (*H*_II_) phase due to a lipid acid configuration that allows the hydrocarbon chains to occupy a larger effective volume. We see our study as an example of a first step in incorporating the insight obtained by combining MD simulations and ML into the design of lipid-based pharmaceutical formulations.

## Supplementary Material



## Data Availability

Data and parameters are openly available at 10.5281/zenodo.11090144. The analysis scripts are available upon reasonable request.

## Notes

Additional description of the APL_vor_ analysis, results on membrane properties, tail conformations and dynamics, and headgroup interactions and dynamics, including assessment of the level of interdigitation (PDF)
