# Supplementary material for: Combination of Molecular Dynamics Simulations and Machine Learning Reveals Structural Characteristics of Stereochemistry-Specific Interdigitation of Synthetic Monomycoloyl Glycerol Analogs
Source: J Chem Inf Model. 2025 Jul 3;65(14):7605–18. doi: 10.1021/acs.jcim.5c00615 (PMC12308798; doi:10.1021/acs.jcim.5c00615)
Supplement: Supplementary file 1 [file ci5c00615_si_001.pdf]

Supplementary Information:

Combination of Molecular Dynamics Simulations and Machine Learning Reveals Structural Characteristics of Stereochemistry-Specific Interdigitation of Synthetic Monomycoloyl Glycerol Analogs

Suvi Heinonen,[*,†] Artturi Koivuniemi,[†] Matthew Davies,[‡,¶] Mikko Karttunen,[‡,¶] Camilla Foged,[§] and Alex Bunker[†]

†*Drug Research Program, Division of Pharmaceutical Biosciences, Faculty of Pharmacy, University of Helsinki, Viikinkaari 5 E, P.O. Box 56, 00014 University of Helsinki, Finland*

‡*Department of Physics and Astronomy, The University of Western Ontario, 1151 Richmond Street, London, Ontario N6A 3K7, Canada*

¶*Department of Chemistry, The University of Western Ontario, 1151 Richmond Street, London, Ontario, N6A 5B7, Canada*

§*Department of Pharmacy, Faculty of Health and Medical Sciences, University of Copenhagen, Universitetsparken 2, 2100 København Ø, Denmark*

E-mail: suvi.p.heinonen@helsinki.fi

Supplementary description of the $\mathrm{APL_{vor}}$ analysis

The $\mathrm{APL_{vor}}$ calculation using the 2D Voronoi tessellation was performed with a Python code implementing MDAnalysis[1] and scipy[2] packages. The lipids were assigned either into upper and lower leaflets by determining the directions of their O1-C35 vectors (see Figure 1 for atom naming). The $\mathrm{APL_{vor}}$ was calculated using the glycerol headgroups (atoms C1, C2, C3, O1, O2, O3). The calculation involved 1) taking the coordinates from the centers of mass (COMs) of the headgroups, 2) finding the nearest neighbors to the COMs within 3×20 Å radius to create neighborhoods that are assumed to be planar 3) calculating the vertices within the neighborhoods and 4) determining the APL using the vertices and the shoelace formula.

Supplementary results

Membrane properties

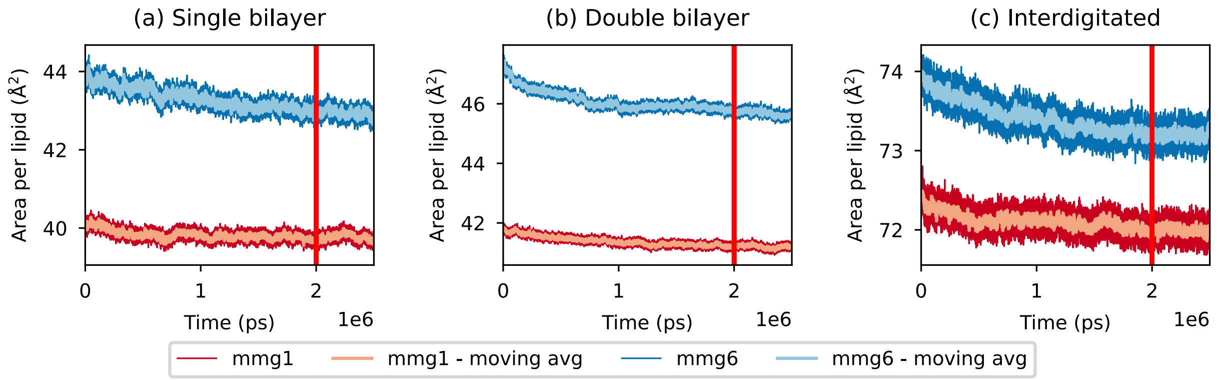

Figure (S1) Area per lipid derived from box size in x and y direction (APL_{xy}) versus time for the (a) single bilayer, (b) noninterdigitated double bilayer, and (c) interdigitated double bilayer systems. The red vertical line denotes where the final 500 ns used for analyses starts. Moving averages have been calculated using 1000 frames.

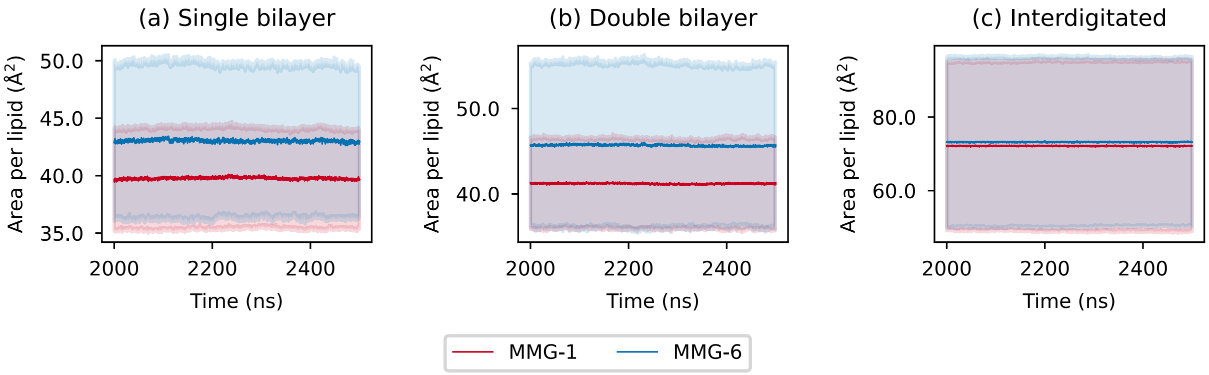

Figure (S2) Area per lipid calculated with 2D Voronoi tesselation (APL_{vor}) versus time for the (a) single bilayer, (b) noninterdigitated double bilayer, and (c) interdigitated double bilayer systems in the final 500 ns of simulation. Average values from the outer leaflets are presented with standard deviations.

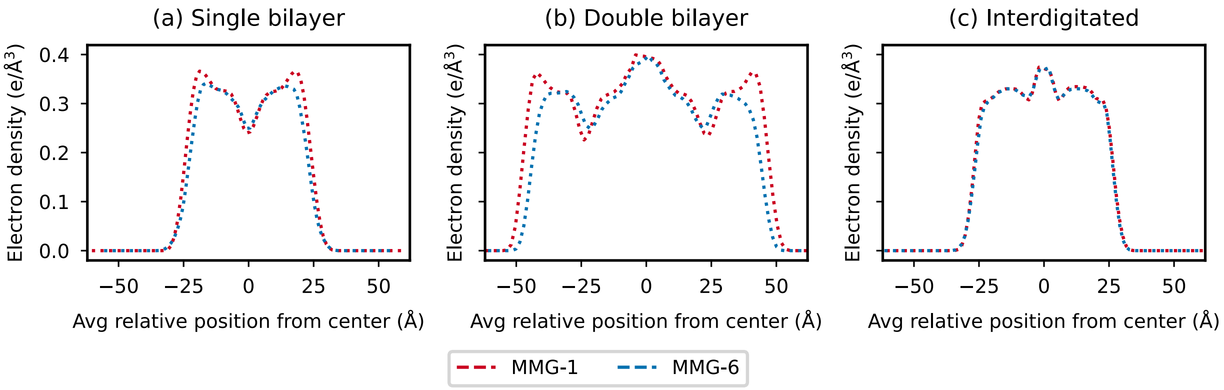

Figure (S3) Electron density profiles of synthetic MMG analogs based on the final 500 ns of simulation. MMG-1 is displayed in red, and MMG-6 in blue. The simulation box has been divided into 100 slices for calculation.

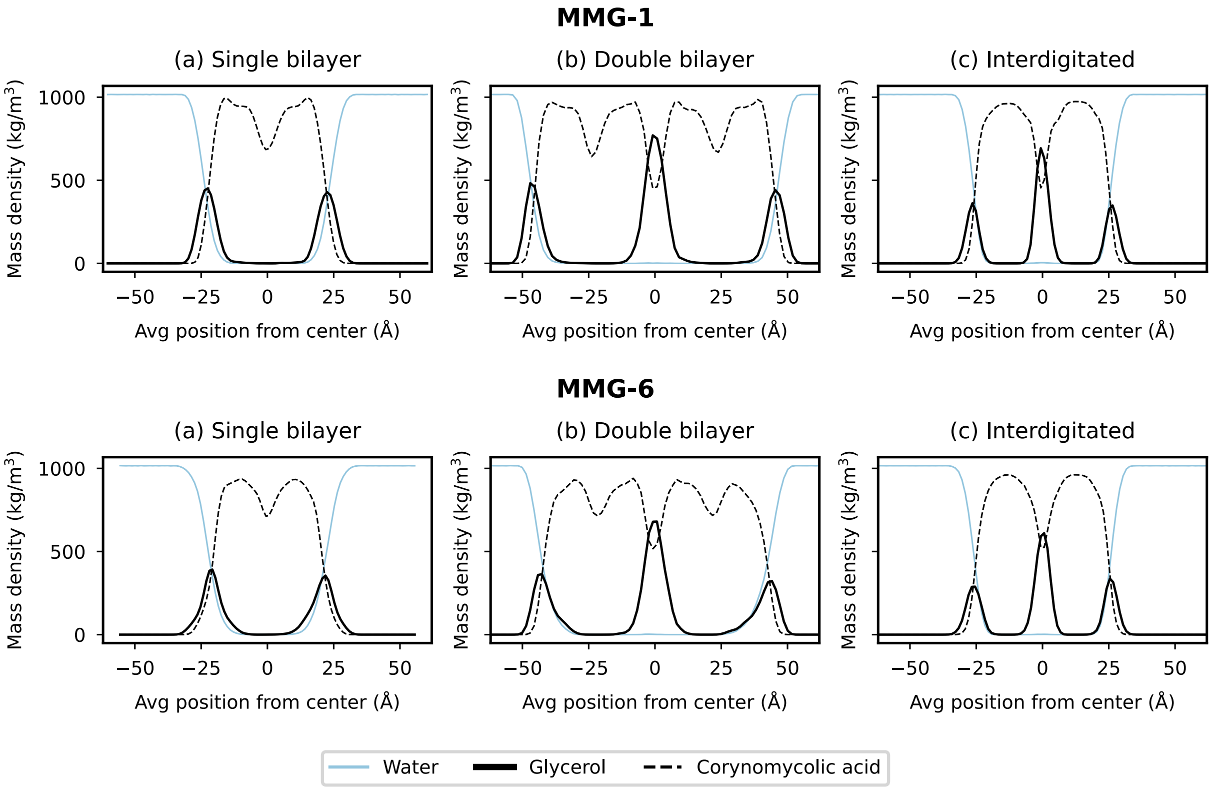

Figure (S4) Mass density profiles of different domains (water, glycerol moiety, and the corynomycolic acid) of the synthetic MMG analogs based on the final 500 ns of simulation. The simulation box has been divided into 100 slices for calculation.

Level of interdigitation

All systems displayed some degree of hydrocarbon tail interdigitation regardless of the MMG analog. Figure S5 shows only the tails in the outer leaflet (or only on one leaflet for the single bilayer system) with the O1 oxygen atoms of the primary hydroxyl groups, thus visualizing interdigitation in the systems. The level of interdigitation was quantified by constructing histograms of the tail carbon atom number density with 120 bins within the range of -60 to 60 Å (Figure S6).

The MMG-1 vs. MMG-6 overlap percentages are 7.9 ± 0.8 % vs. 12.1 ± 1.0 % for single bilayers (Figure S6a,d), 7.2 ± 0.4 % vs. 12.8 ± 0.4 % for double bilayers (Figure S6b,e), and 78.1 ± 0.2 % vs. 78.3 ± 0.2 % for the interdigitated bilayers (Figure S6c,f). The overlap percentages and standard deviations were calculated using the carbon atoms in the hydrocarbon chains based on the final 500 ns of simulation. Overall, MMG-6 displays a larger histogram overlap percentages in the single and double bilayer systems.

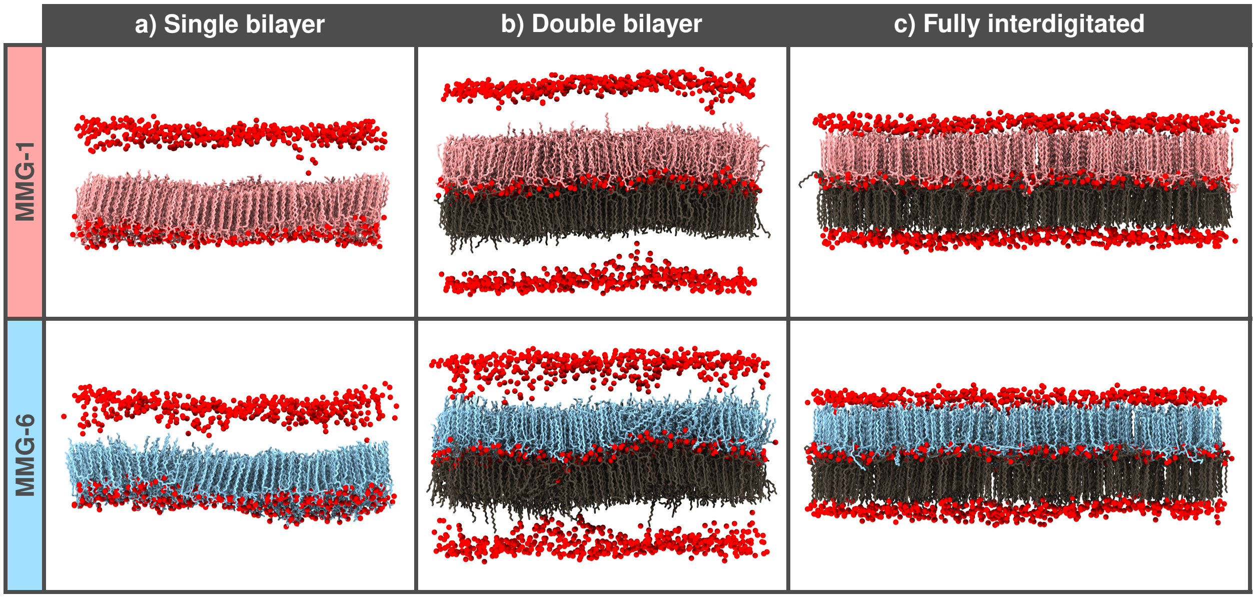

Figure (S5) Visualization of tail interdigitation in noninterdigitated membranes at t = 2,500 ns. Hydrocarbon tails of the inner leaflet MMG molecules have been omitted to display tails extending to the opposite leaflet. MMG-1 is colored pink and dark taupe, and MMG-6 is colored blue and dark taupe. The oxygen atoms of the primary hydroxyl group are displayed in red.

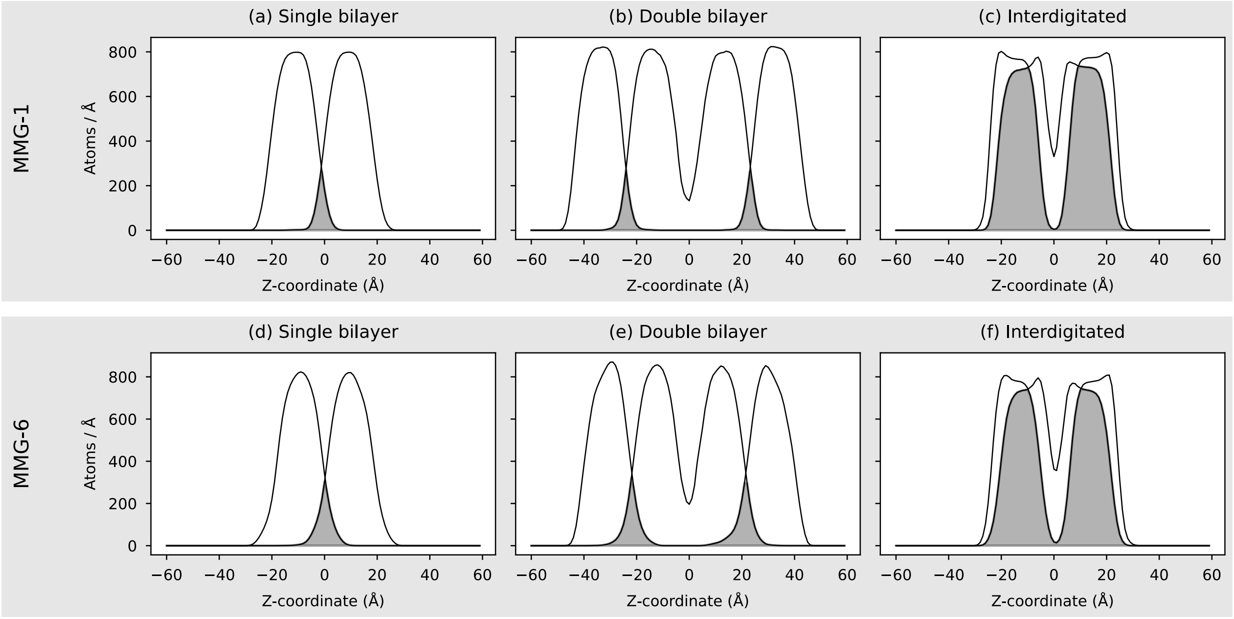

Figure (S6) Average number density profiles of MMG tail atoms over the final 500 ns of simulation. The overlap has been colored gray.

Supplementary results on tail conformations and dynamics

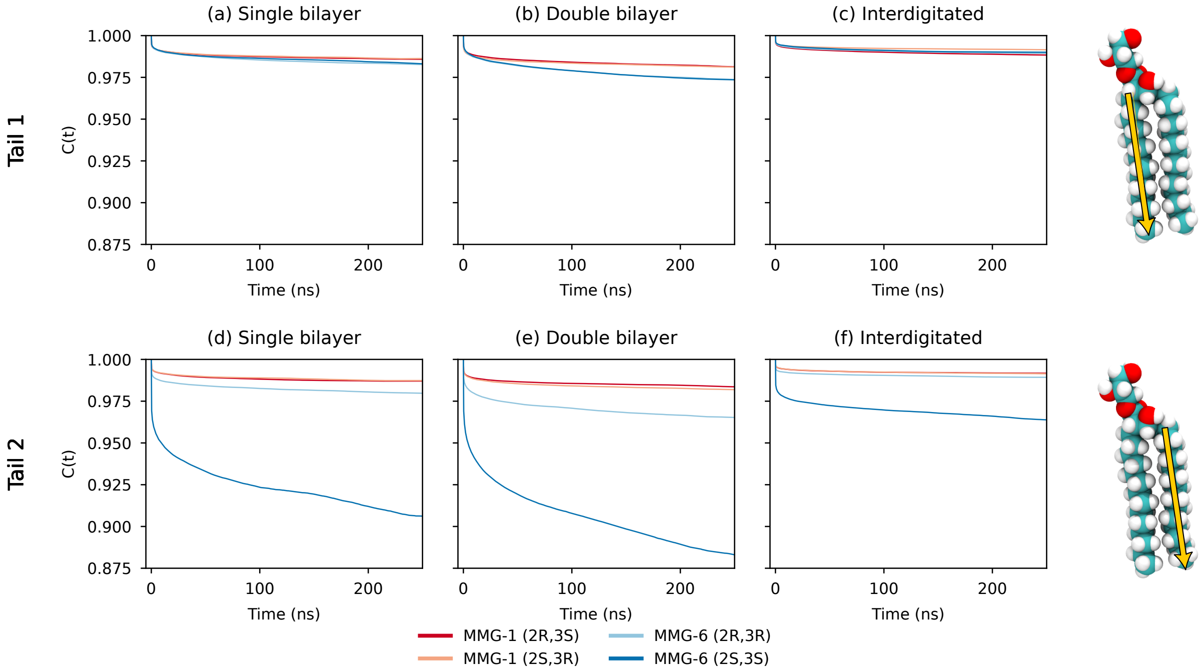

Figure (S7) Rotational autocorrelations of the tail vectors: tail 1 (C7-C20) (a,b,c), and tail 2 (C21-C35) (d,e,f). See Figure 1 for atom numbering.

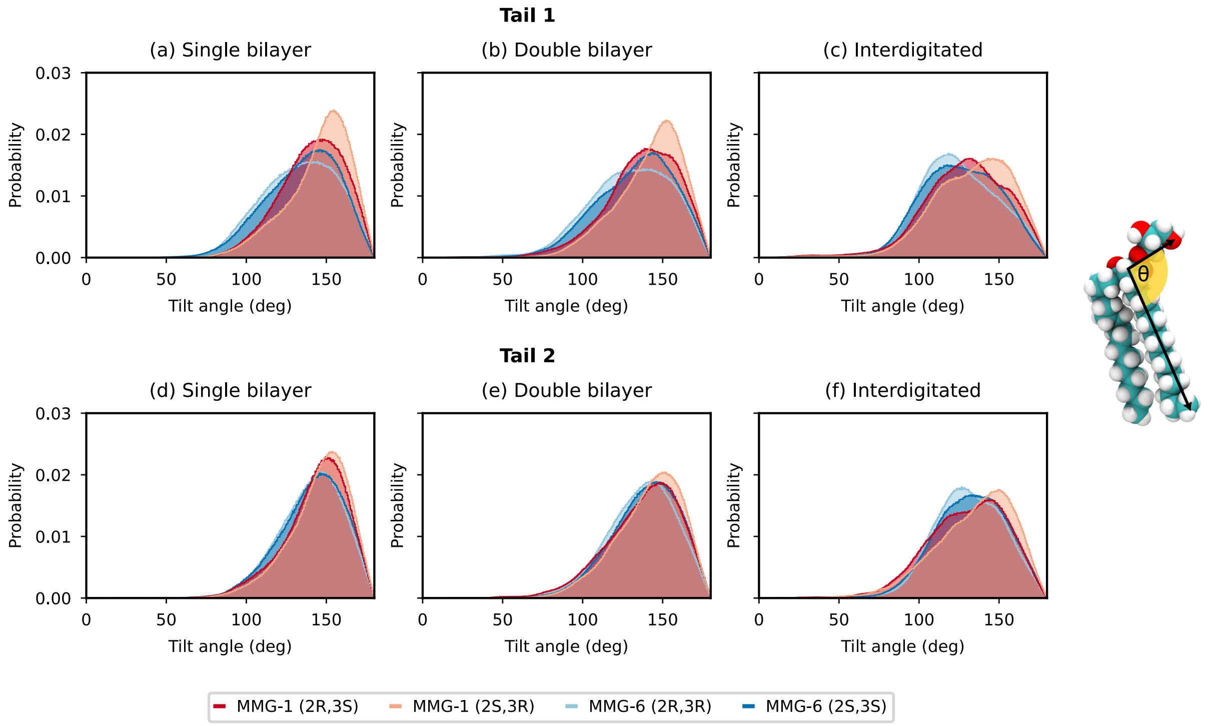

Figure (S8) Probability distributions for the angle between the MMG headgroup vector (C5-O1) and the hydrocarbon tail vectors C5-20, C5-C35. See Figure 1 for atom numbering.

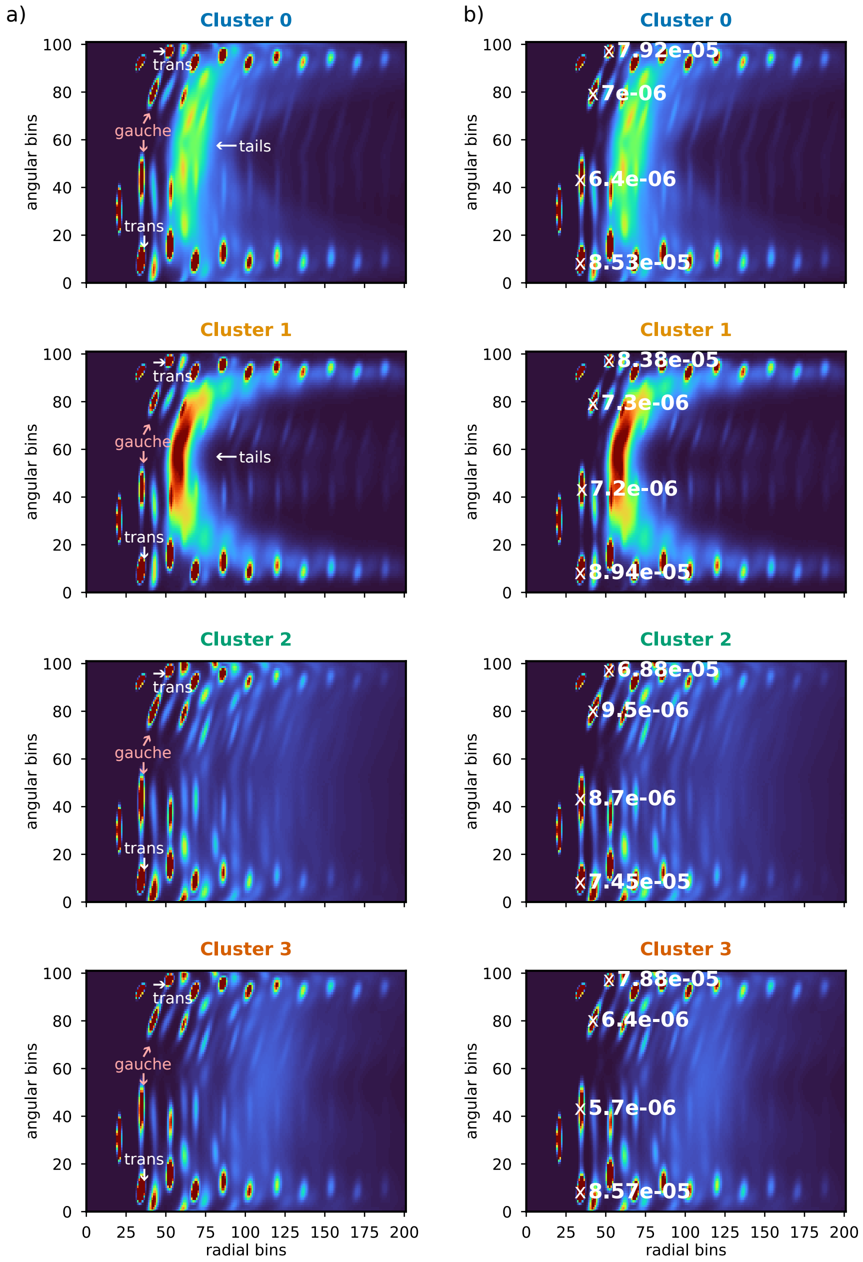

Figure (S9) Average g_3 distributions with explanations (a) and annotated peaks (b). The trans/gauche peak ratios calculated from the corresponding peaks for each cluster are 13.3, 11.3 (cluster 0); 12.4, 11.5 (cluster 1); 8.6, 7.2 (cluster 2) and 15.0, 12.3 (cluster 3). The ratios have been calculated for the atoms on the same and the opposite side from the central atom separately.

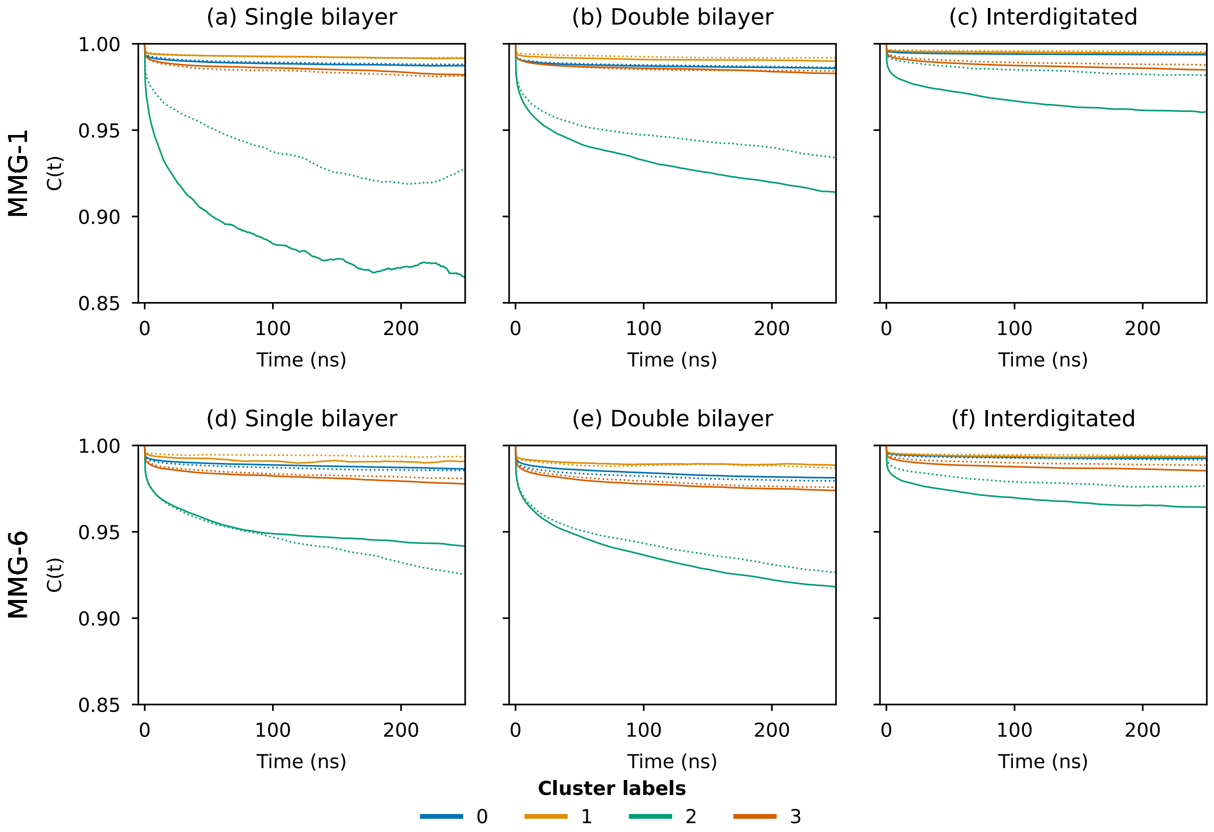

Figure (S10) Rotational autocorrelation functions of the tail vectors (C7-C20, C21-C35). The autocorrelation functions have been analyzed separately for MMG clusters found through the g3 cluster analysis. Tail 1 is represented by a solid line, and tail 2 by a dashed line.

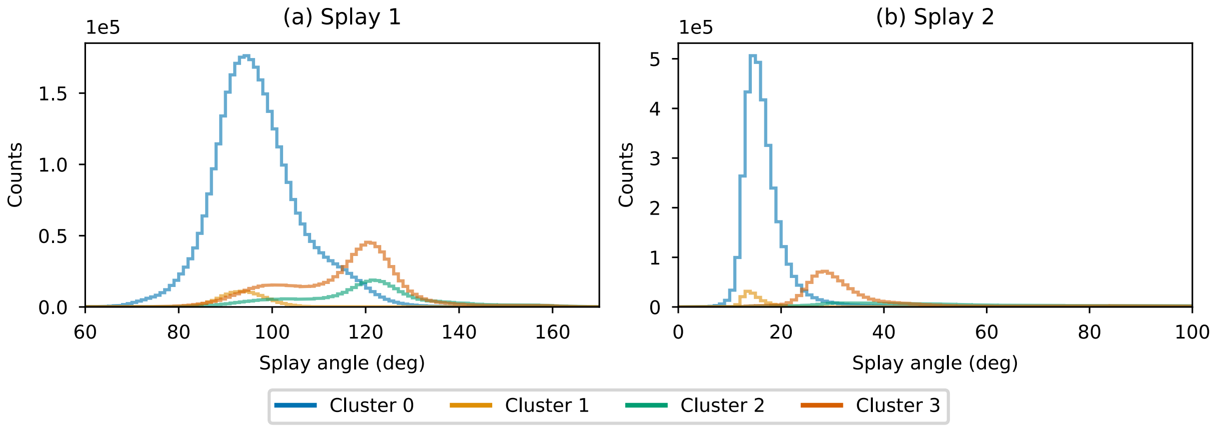

Figure (S11) Splay angles counted in each of the clusters found through the g3 analysis. The analysis has been carried out using the final 500 ns of the simulation trajectory (5000 frames). The splay angles in this figure were calculated using MDAnalysis. GROMACS tools were used for obtaining the splay angle results in the main text.

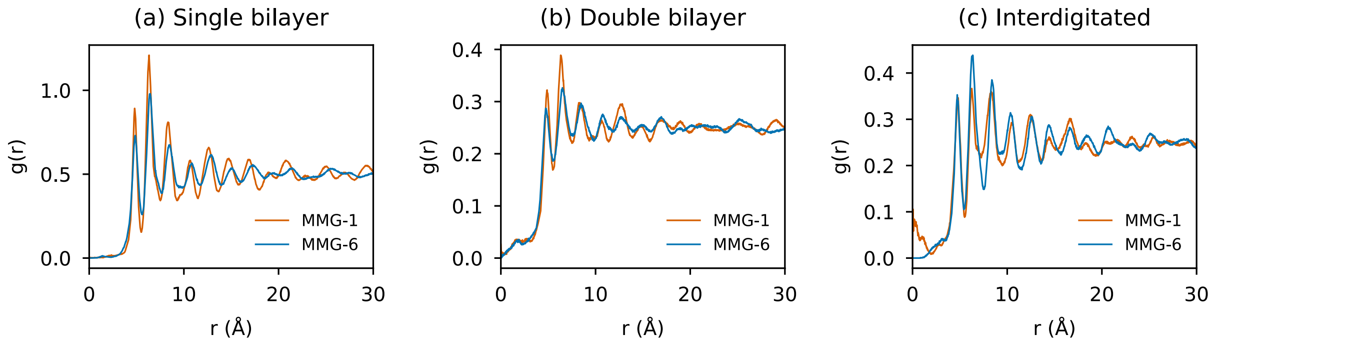

Figure (S12) Average 2D radial distributions of MMG tail centers of mass as a function of distance from other MMG tail centers of mass. The radial distribution functions have been calculated separately for each leaflet and then averaged.

Supplementary results on headgroup interactions and dynamics

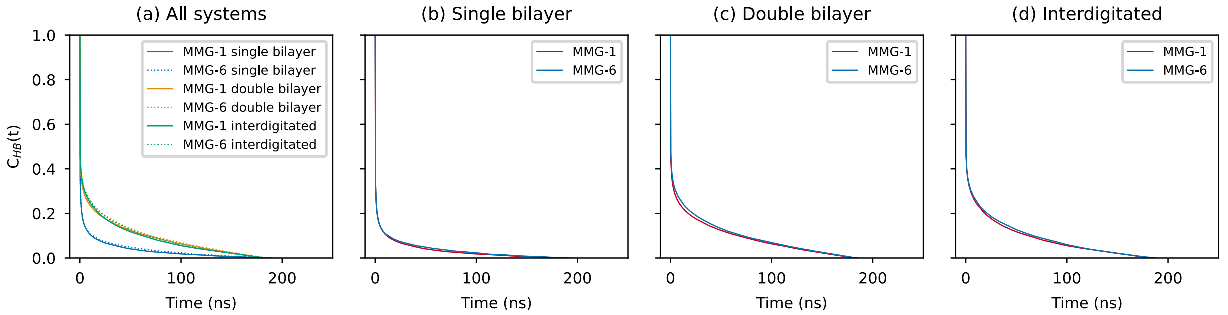

Figure (S13) Tail-corrected hydrogen bond autocorrelation functions for a) All systems, b) single bilayer, c) double bilayer, and d) interdigitated cases. Both intra- and intermolecular hydrogen bonding between the MMG molecules has been considered in the calculation. The autocorrelation functions have been calculated based on the final 500 ns of simulation.

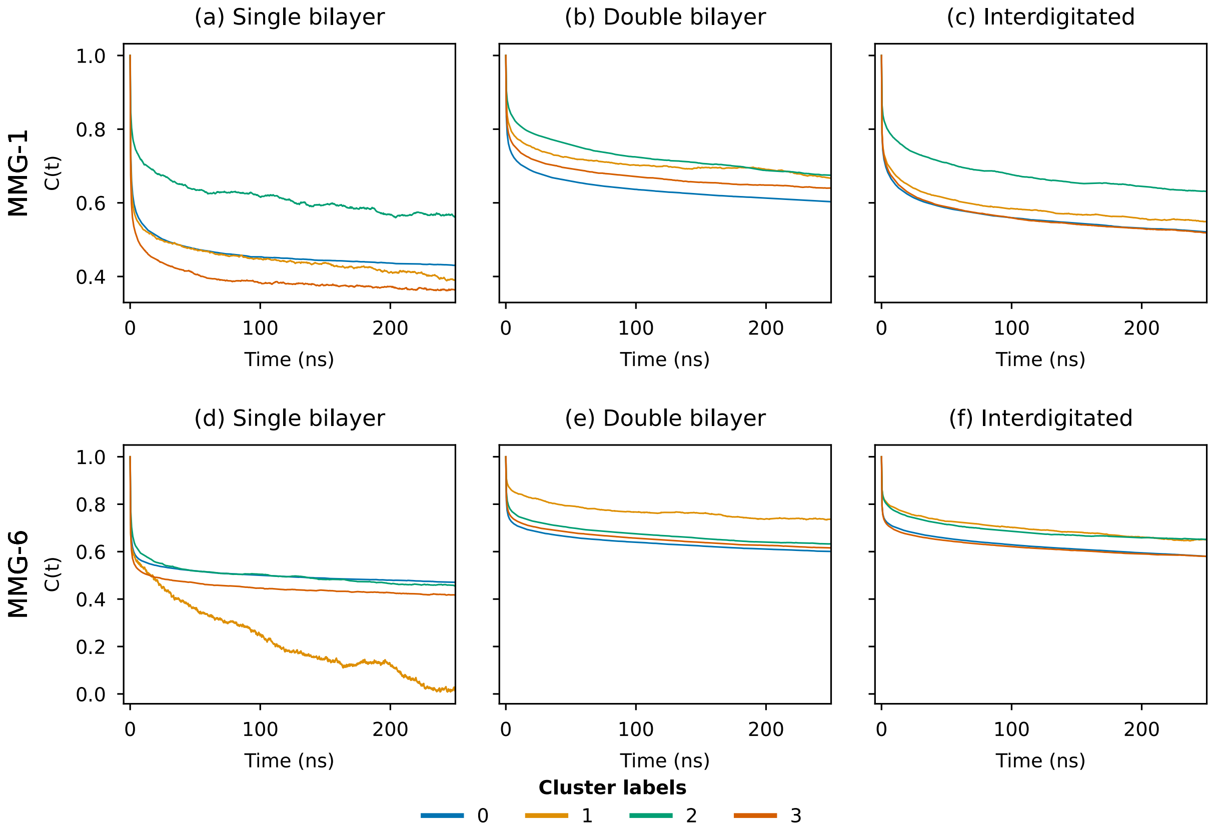

Figure (S14) Rotational autocorrelation functions of the glycerol moiety C1-C3 vectors, see atom numbering in Figure 1. The autocorrelation functions have been analyzed separately for MMG clusters found through the $g3$ analysis.

References

(1) Michaud-Agrawal, N.; Denning, E.; Woolf, T.; Beckstein, O. MDAnalysis: A toolkit for the analysis of molecular dynamics simulations. *J. Comput. Chem.* **2011**, *32*, 2319–2327 .

(2) Virtanen, P.; Gommers, R.; Oliphant, T. E.; Haberland, M.; Reddy, T.; Cournapeau, D.; Burovski, E.; Peterson, P.; Weckesser, W.; Bright, J.; van der Walt, S. J.; Brett, M.; Wilson, J.; Millman, K. J.; Mayorov, N.; Nelson, A. R. J.; Jones, E.; Kern, R.; Larson, E.; Carey, C. J.; Polat, İ.; Feng, Y.; Moore, E. W.; VanderPlas, J.; Laxalde, D.; Perktold, J.; Cimrman, R.; Henriksen, I.; Quintero, E. A.; Harris, C. R.; Archibald, A. M.;

Ribeiro, A. H.; Pedregosa, F.; van Mulbregt, P.; SciPy 1.0 Contributors. SciPy 1.0: Fundamental Algorithms for Scientific Computing in Python. *Nat. Methods* **2020**, *17*, 261–272.
